# Toward Digital Self-Monitoring of Mental Health in the General Population: Scoping Review of Existing Approaches to Self-Report Measurement

**DOI:** 10.2196/59351

**Published:** 2025-09-18

**Authors:** Zhao Hui Koh, Duygu Serbetci, Jason Skues, Greg Murray

**Affiliations:** 1 Centre for Mental Health and Brain Sciences Swinburne University of Technology Hawthorn Australia; 2 Department of Psychological Sciences Swinburne University of Technology Hawthorn Australia

**Keywords:** mental health, digital health, monitoring, repeated measurement, general adult population, self-report instrument, scoping review, mobile phone

## Abstract

**Background:**

With the ubiquity of smartphones, digital self-report instruments have enormous potential to support the general population in monitoring their mental health. A primary challenge for researchers committed to advancing this work is simply to scope the plethora of widely used candidate instruments. The overarching aim of this study was to address this challenge to support and guide future research in this burgeoning area.

**Objective:**

This study aimed to conduct a literature review of self-report instruments used in empirical studies to measure mental health (1) in the general population, (2) delivered in a digital format, and (3) in longitudinal designs. Given the wide range of recognized “mental health” constructs, the review’s search strategies were guided by Keyes’ dual continua model of mental health, recognizing both deficits- and strengths-based constructs. This study’s primary objective was to develop a first-of-its-kind ranking and synthesis of the most frequently used instruments that are potentially suitable for mental health self-monitoring. It was not an objective of this study to evaluate psychometric properties of the identified instruments—we hope the present ranking and synthesis will provide the foundation for future research into optimal digital, prospective self-report of mental health.

**Methods:**

Five major electronic databases were searched. Studies that administered digital mental health instruments (in English) repeatedly to community dwellers in the general adult population were eligible. The included studies were grouped by instruments for synthesis using a narrative approach.

**Results:**

Preliminary screening of 95,849 records identified 8460 eligible records, among which 1000 records were randomly selected over 4 iterations for full-text screening. A total of 223 records were included. We found that the top 30 most commonly used instruments accounted for 78.4% (308/393) of the total usage across studies. These instruments predominantly measure deficits-based mental health constructs. The Patient Health Questionnaire 9 Items and Generalized Anxiety Disorder 7 Items were by far the most used instruments. The most commonly measured strengths-based constructs were life satisfaction and mental well-being.

**Conclusions:**

The findings of this review strongly suggest that scientific investigation of mental health constructs across time on digital platforms still prioritizes deficits-focused instruments originally developed for pen-and-paper administration using classical test theory. These findings are discussed in light of evidence in the literature that deficits-focused instruments demonstrate inferior distributional properties (floor effects) in the general population and theory suggesting that both deficits- and strengths-focused measurements are required to holistically assess mental health. Limitations of the review include the restricted focus on English language instruments and the pragmatic approach to selecting records for full-text screening. It is concluded that, in the smartphone age, it would be timely to develop new digital instruments framed by holistic models of mental health and using contemporary test construction approaches.

**Trial Registration:**

PROSPERO CRD42022306547; https://www.crd.york.ac.uk/PROSPERO/view/CRD42022306547

**International Registered Report Identifier (IRRID):**

RR2-10.1136/bmjopen-2022-065162

## Introduction

### Background

As mental health researchers with a digital interest, we are increasingly approached by researchers and industry partners to recommend self-report instruments that can be offered to the general public for monitoring mental health digitally. A barrier to providing an evidence-based answer to this apparently simple question is the lack of an overview of instruments that have already been used for this or related purposes. The overarching aim of this review, consequently, was to scope existing instruments that could fulfill this role, as demonstrated by their use in published empirical research. Broadly, we sought to answer the question: which instruments have been most frequently used, and what are their characteristics? First, we briefly review evidence that digital technologies render self-monitoring more feasible than ever before, and introduce contemporary approaches to test construction. We then introduce the dual continua model of mental health that was used to guide the systematic search strategy and results synthesis of this study.

### Self-Monitoring of Mental Health

Self-monitoring has proven effective and economical in managing many chronic illnesses [1-6], and is potentially a useful component of population-based approaches to managing mental health [7-10]. In the realm of mental health, self-monitoring via self-report inventories can raise emotional self-awareness [11] and promote positive health behavioral change over time [12]. Self-monitoring mental health can detect early symptoms of mental illness [13] while also providing a better understanding of factors that influence positive mental health [14-16], aligning with the goals of mental illness prevention and mental health promotion programs [17]. The focus here was on the full range of mental health constructs that have been measured by self-report, including general mental health measures (eg, the Kessler Psychological Distress Scale [K10] [13]), and instruments designed to screen for (eg, the Patient Health Questionnaire 9 Items [PHQ-9] [18]), or monitor severity of diagnosable disorders (eg, the Beck Depression Inventory II [19]).

Ubiquitous digital platforms have enormous potential to support self-monitoring of mental health in the general population. These opportunities are increasingly recognized in fields including digital monitoring [20], digital assessments [21], digital phenotyping [22], and self-management [3]. Remarkably, it is estimated that more than 10,000 mobile apps are available in the digital mental health market [23,24], and common mental illnesses such as anxiety and depression are measured in numerous associated apps [25-28]. Importantly, these apps typically incorporate existing validated self-report instruments in a digital format for self-tracking purposes [11]. For example, the PHQ-9 is often used as a measure of depressive symptoms, despite being developed in the predigital era.

A shift toward digital modalities of data collection also supports a transition in survey development from classical test theory (CTT) to item response theory (IRT). CTT [29] has been the dominant approach to survey development for traditional paper-and-pen surveys. However, advances in measurement theories such as IRT [30,31] highlight several shortcomings of CTT and are starting to positively influence psychological test development through improved measurement precision [30,32,33]. One notable application of IRT, originating from educational assessments, is the Computerized Adaptive Test (CAT) [34], which harnesses the computational power of digital technologies to provide dynamic survey administration. CAT can maintain the precision of mental health measurement while significantly reducing the total number of questions and thereby alleviating the response burden [35,36]. A prominent example of this contemporary approach to measurement is the PROMIS (Patient Reported Outcome Measures Information System), a program initiated by the US National Institutes of Health [37]. PROMIS includes suites of mental health outcome measures designed with digital administration in mind, available in various formats such as fixed-length (eg, comprehensive long-form or equivalent short-form self-report assessments) and computerized adaptive testing assessments.

### Dual Continuum Model of Mental Health

Although originally linked to mental illness diagnoses [38-41], contemporary theorizing [14,42-50] conceptualizes mental health as multifaceted, subsuming deficits-based constructs related to mental illness, but also strengths-based constructs such as subjective well-being [51], the mental health continuum [52], and flourishing (in positive psychology) [53].

**Figure 1:**
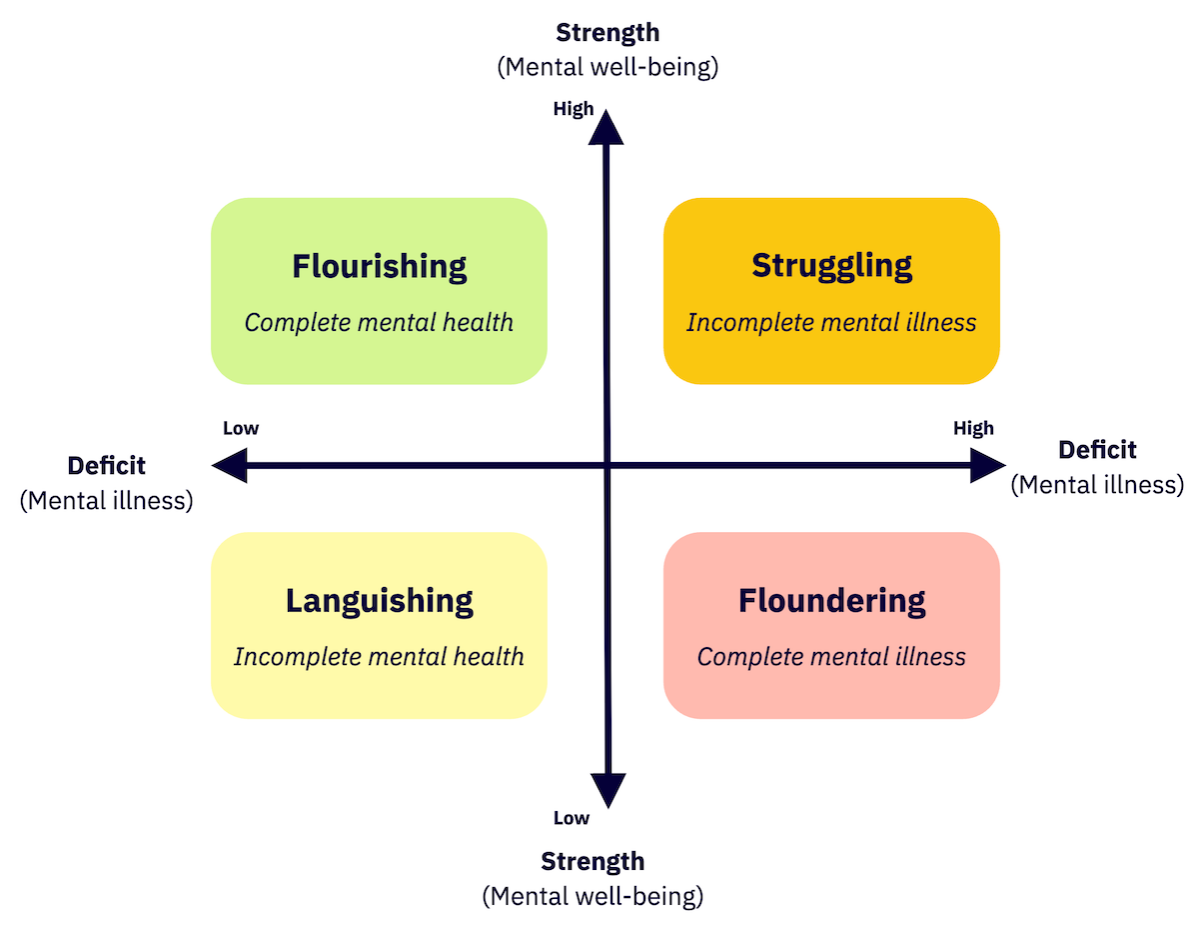
Dual continua model of mental health [14] (adapted from Teng et al [57] with permission).

The influential dual continua model of mental health [14] recognizes 2 correlated but separable dimensions, typically named “mental illness” and “mental well-being” (for clarity, we use the terms “deficit” and “strength” here [54], see Figure 1). This conceptual model explains more variance in diverse populations than an alternative model in which mental illness and mental well-being are placed at opposite ends of a single continuum [55]. As described in the protocol paper for this study, the dual continua model directly informed the present search strategy and the synthesis of findings (Multimedia Appendix 1 [56]).

### This Review

The present review is unique because it intends to advance understanding of the range of instruments that could function as mental health self-monitoring tools in the digital era. Specifically, we systematically searched for empirical studies that used instruments measuring mental health in (1) the general population, (2) digital format, and (3) longitudinal designs. While there is a growing body of research investigating measures of mental health in the nonclinical adult population [58-63], there is a lack of reviews that focus specifically on studies that meet the 3 aforementioned criteria. Consequently, this review has a narrow focus on instruments that have been applied in a manner comparable to digital self-monitoring of mental health. To the best of our knowledge, no review shares the specific focus of the present review. We anticipate that the review findings will be useful to the burgeoning number of researchers who share our interest in valid digital self-report measurements of mental health in the general population [59,64].

The aim of this scoping review was therefore to systematically identify empirical studies that used instruments measuring mental health (broadly defined) in (1) the general population, (2) digital format, and (3) longitudinal designs. This aim was addressed with the following research question (RQ):

RQ: Which self-report mental health instruments have been most frequently used in empirical studies via digital delivery to measure the mental health of the general adult population at more than 1 time point?

In this review, usage of the instrument is operationalized as the frequency of the instrument being administered in identified empirical studies over the number of years since the release of the instrument, bounded by the time frame of the review (ie, papers published between January 1, 2010, and December 31, 2021, inclusively).

The peer review process has led to some changes to study reporting that represent deviations from the original study protocol [56]. Most importantly, the study was originally designed as a systematic review, but through peer review, the authors learnt that it is easier to understand the study as a scoping review with a systematic literature review step. Second, we have reorganized the presentation of this study’s RQs as described in the protocol. The primary RQ above is a more succinct wording of the erstwhile questions RQ1 and RQ4. To avoid misunderstanding about their status in the study design, ancillary questions about the characteristics of the most popular instruments (erstwhile RQ2, RQ3, and RQ5) have been removed from the main text (see Multimedia Appendix 2), and the findings now appear only in Section S2 in Multimedia Appendix 3 [13,14,16,18,19,56,64-237].

## Methods

### Study Design

This scoping review adheres to the PRISMA-ScR (Preferred Reporting Items for Systematic Review and Meta-Analysis Extension for Scoping Reviews) 2018 guidelines [238]. Methods such as eligibility criteria, information sources, search strategy, selection process, data collection process, data extraction, risk of bias (quality) assessment, and data synthesis plan are described in our published protocol [56]. The following section elaborates on deviations from the original protocol and the execution of these methods. Section S1 in Multimedia Appendix 3 presents the full search terms and strategies used for all databases in this review.

### Selection Process

#### Eligibility Criteria

In the original protocol [56], eligibility criteria were defined in the Population, Intervention, Comparison, Outcome, and Time (PICOT) [239] format. For this scoping review, a mapping from the PICOT format to the population, concept, and context [240] format is detailed below. The population is identical to the protocol. The concept represents the types of empirical studies included and the scope of the mental health constructs considered, which were associated with the section “Intervention of Interest” of the protocol [56]. The context in this review represents the timeframe of the included published papers, which is between January 1, 2010 and December 31, 2021 (inclusive).

#### Preliminary Screening of Titles and Abstracts

In a preliminary scoping of the literature, a large number of search results (over 90,000 records) were returned, requiring the application of various tools to help screen records. One reviewer (ZHK) screened the titles and abstracts of the search records using text-mining techniques [241]. Subsequently, the screening effort was validated using a semiautomated screening tool (ASReview [242]). The details of this preliminary screening process are described elsewhere [243].

#### Full-Text Screening

A large number of eligible records (N=8460) were identified for full-text review after preliminary screening. This large record count is a result of our broad interest in the most frequently used mental health instruments being administered by past empirical studies. Furthermore, our three inclusion criteria (instruments being used in (1) the general population, (2) digital format, and (3) across multiple time points) are often not mentioned in the records’ title or abstract, as they relate more to methods than the substantive questions of the study.

Considering that a typical review takes, on average, 5 members and approximately 67.3 weeks to conduct and publish [244,245], full-text screening of 8460 records was not feasible with 2 reviewers and limited resources. This feasibility challenge has been acknowledged by COnsensus-based Standards for the selection of health Measurement INstruments (COSMIN) when reviews are conducted on patient-reported outcome measures [246]. The large return of eligible studies, therefore, forced us to develop a pragmatic approach to full-text screening. Previous systematic reviews facing similar challenges, for example, these studies [247-251] have screened a subset of the eligible records using either a random sampling strategy (eg, stratified sampling by time period) or a data saturation strategy to ensure the representativeness of the variables of interest.

Here, we also decided to screen a subset of eligible records, but adopted a statistical strategy to test whether or not the subset was representative of the entire population of eligible records. We randomly selected a subset of 1000 records from the 8460 records eligible for full-text screening in batches of 250 (no repeats) over 4 iterations of sampling. As the primary focus of the present review was instruments’ usage frequency (above), usage frequency was the variable used to statistically investigate the representativeness of the subset of n=1000 records. It was hypothesized that if the distribution of the usage frequency of instruments in each of the 4 batches was not statistically different, given the 4 batches were randomly selected from the same population of 8460 records, it could be inferred that the distribution of the usage frequency of instruments in each batch was likely to reflect in the full set of 8460 records [252-256]. The hypothesis was tested using statistical techniques (chi-square test of homogeneity [257] and Fisher exact test [258], significance level α of .05) once the 1000 records were screened in full-text and the data were extracted from the included records.

**Figure 2:**
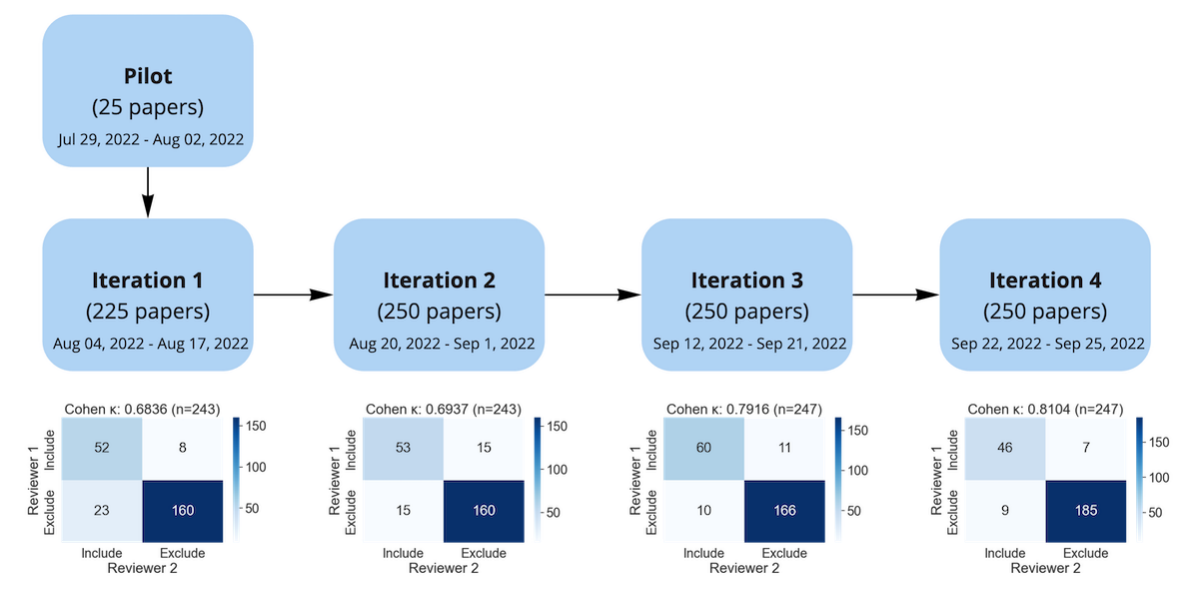
Full-text screening process of 1000 randomly sampled records in 4 iterations.

Informed by these approaches, 2 reviewers (ZHK and DS) screened the selected 1000 records in 4 iterative batches of 250 (Figure 2). To help manage the screening process, we developed a tool [259] to facilitate full-text screening and data extraction (hereafter referred to as “the custom tool”). Both reviewers pilot-tested the custom tool with 25 randomly selected records before commencing the full-text screening and data extraction. In each iteration, both reviewers independently screened 250 records in full text. If the record was still eligible, relevant data were extracted using the custom tool. If further information was required, ZHK contacted the researchers of the studies. After each iteration, both reviewers reconciled the review results and resolved any disagreements. The interrater agreement between reviewers was calculated using Cohen κ [260] (Figure 2). Suggestions to improve the custom tool were considered and implemented in subsequent iterations.

After 2 iterations, it became apparent that one of the main disagreements between reviewers was determining whether the instruments used in these records were in English, as not all records explicitly declared the language of the instruments. A decision flowchart was created (Multimedia Appendix 4) to facilitate the decision-making between reviewers after iteration 2. Since then, the interrater agreements were improved in iteration 3 and were maintained in iteration 4.

### Data Extraction Process

Both reviewers (ZHK and DS) independently extracted data from each record using a separate copy of the custom tool. After completing each iteration, ZHK reconciled the instruments extracted from both reviewers. Then, both reviewers discussed any discrepancies and resolved disagreements before commencing the next iteration.

### Quality Assessment

While the usage frequency of instruments is the main focus of the present scoping review, to ensure this review is useful to future researchers, similar to past reviews [64-66], we briefly commented on the quality assessment at the instrument level instead of individual studies, with a provisional evaluation of their core psychometric properties using studies from secondary searches (Section S2 in Multimedia Appendix 3).

### Data Synthesis

Findings are summarized using a narrative synthesis approach [261] guided by the Synthesis Without Meta-Analysis (SWiM) reporting guideline [262] and framed by Keyes dual continua model (above). As described in our published protocol [56], the included studies were grouped by instruments and synthesized in tabular format, summarizing the properties of all studies associated with each instrument. The usage of instruments among the included studies is presented descriptively as figures.

### Ancillary Information

This study was not designed to rank identified instruments in terms of psychometric properties. It is expected that the present frequency-of-use ranking and synthesis will provide the foundation for future researchers to ask questions about the optimal approach to digital, prospective self-report of mental health (eg, selecting the best instrument for a particular population or building new adaptive instruments from item content in established instruments). For each instrument identified, ZHK manually searched the literature to elicit the ancillary information, such as the instrument’s structure, format, and psychometric properties. The ancillary information was then synthesized in tabular format (Section S2 in Multimedia Appendix 3). The psychometric properties of each instrument were provisionally rated based on the quality criteria defined by Terwee et al [67].

## Results

### Overview

**Figure 3:**
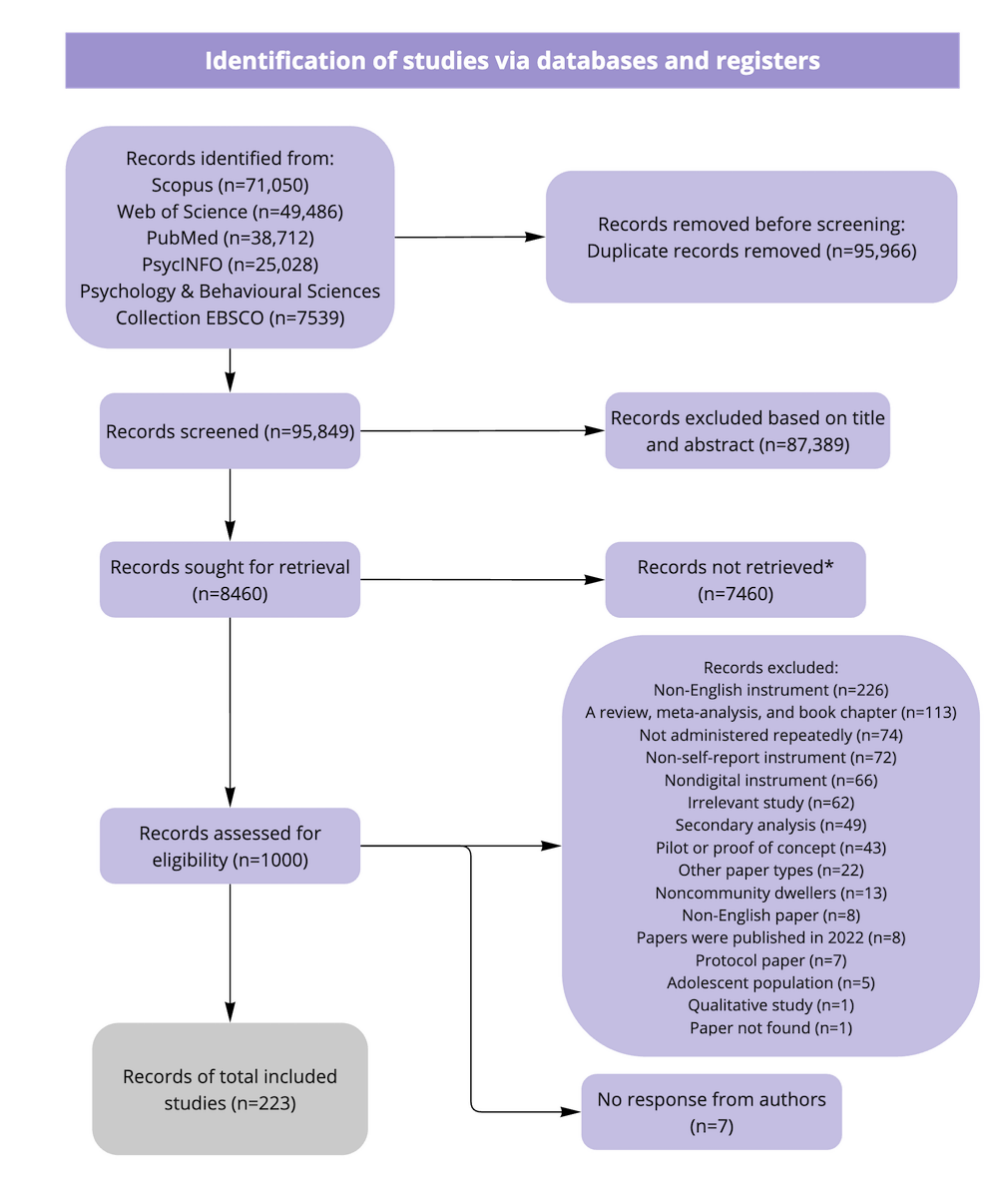
PRISMA (Preferred Reporting Items for Systematic Reviews and Meta-Analyses) flowchart showing how records were identified from the initial search to the final decision. *: for pragmatic reasons, 1000 records were randomly sampled from 8460 records over 4 iterations for full-text screening. Statistical testing of the validity of this pragmatic strategy is described above and reported below.

Searches on 5 databases (Scopus, Web of Science, PubMed, PsycINFO, and Psychology & Behavioral Sciences Collection [via EBSCOhost]) returned 191,815 records, of which 95,849 were unique records after deduplication (Figure 3). Preliminary screening of titles and abstracts resulted in 8460 eligible records for full-text screening. As noted above, 1000 of 8460 eligible records were randomly selected in batches of 250 for full-text screening over 4 iterations. Statistical tests were conducted to evaluate the representativeness of the instruments’ usage distribution (the key variable of interest) within each iteration across the 1000 records, as described in the results below. Among these 1000 records, 770 were deemed ineligible, and 7 records were excluded through author nonresponse to queries from ZHK. A representative group of 223 records was included for data extraction and synthesis.

Among the 770 records deemed ineligible during full-text screening, the most common reasons for excluding records were that the instrument was non-English [263-265], records were reviews, meta-analyses, or book chapters [25,266], the instrument had not been administered repeatedly [267,268], was not self-reported [269,270], or was nondigital [271,272].

**Figure 4:**
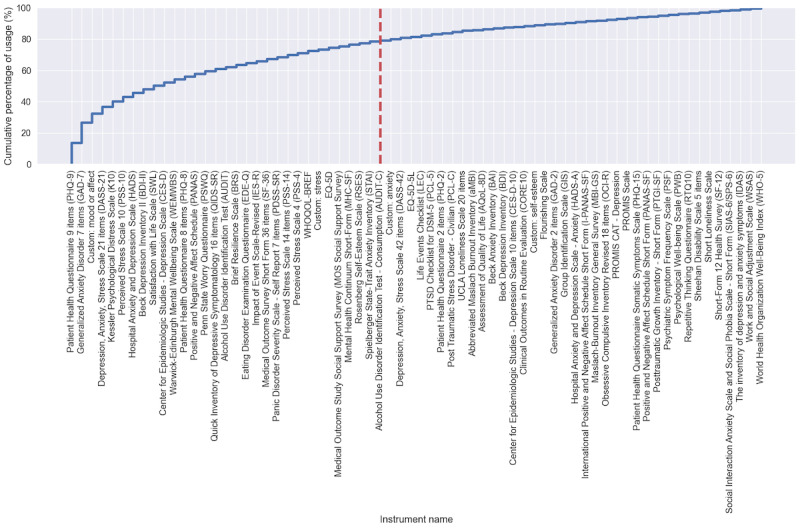
Cumulative percentage of the 68 instruments’ usage across studies. Thirty instruments were selected for synthesis (left of the red dotted line) and accounted for 78.4% (308/393) of the total instruments’ usage across studies. Instruments with names prefixed with “custom:” are groups of custom questions created by researchers to measure similar mental health constructs commonly used in ecological momentary assessment studies. For example, the instrument named “custom: mood or affect” includes custom questions measuring mood or affect.

The 223 included records (empirical studies) reported the usage of 278 eligible self-report mental health instruments, of which 68 were used in more than 1 record. To ensure our synthesis was manageable, we selected instruments that were most commonly used across studies, resulting in 30 of 68 instruments, which accounted for approximately 78.4% (308/393) of the total instruments’ usage across studies (see Figure 4 for details about the selection).

Six instruments in Figure 4 were excluded from the 30 instruments to narrow the measurement construct more precisely onto states of mental health. Specifically, instruments were excluded for the following reasons: first, the Penn State Worry Questionnaire, Brief Resilience Scale, and Rosenberg Self-Esteem Scale were excluded because they measure traits, which are not the focus of this review. These instruments were not excluded during screening and data extraction because it was unclear that they measured trait-like constructs based on their repeated usage in the included empirical studies. It became apparent that subsequent secondary searches that obtained the original publication of these instruments have illuminated the ineligibility of these instruments in this review (n=3). Second, the MOS Social Support Survey measures social support rather than mental states (n=1). Third, custom questions on mood or affect and stress were excluded because they were nonstandardized instruments, with question items varying from study to study (n=2). The synthesis from here on will focus only on the remaining 24 instruments (Table 1) among 147 studies.

The statistical investigation of our pragmatic strategy for reducing the number of records for full-text review involved, first, a chi-square test of the distribution of instruments’ usage across the 4 iterations of sampling. As expected, the test was not significant (*χ*^2^_69_=60.98, *P*=.74). However, one of the assumptions (80% of the expected values >5) of the chi-square test was not met. Hence, a Fisher Exact Test was subsequently conducted, which was also not significant (*P*=.74). Therefore, we could not reject the null hypothesis of no difference, indicating that the usage distribution of instruments across batches was not significantly different. Given that each batch of records was randomly sampled from a population of 8460 records, this finding suggests that the usage distribution of instruments presented in these batches most likely approximates the overall instrument usage of all eligible records.

**Table 1:** The 24 instruments selected for synthesis, sorted by descending usage frequency.

Instrument	Mental health constructs measured	Deficits- or strengths-focused
Patient Health Questionnaire 9 Items (PHQ-9) [18]	Depression	Deficits
Generalized Anxiety Disorder 7 Items (GAD-7) [68]	Anxiety (generalized anxiety disorder)	Deficits
Depression, Anxiety, Stress Scale 21 Items (DASS-21) [69]	Depression, anxiety, and stress	Deficits
Kessler Psychological Distress Scale (K10)^a^ [13]	Psychological distress	Deficits
Perceived Stress Scale 10 Items (PSS-10) [70,71]	Stress	Deficits
Hospital Anxiety and Depression Scale (HADS) [72]	Anxiety and depression	Deficits
Beck Depression Inventory II (BDI-II) [19]	Depression	Deficits
Satisfaction With Life Scale (SWL) [73]	Life satisfaction	Strengths
Center for Epidemiologic Studies-Depression Scale (CES-D) [74]	Depression	Deficits
Warwick-Edinburgh Mental Well-Being Scale (WEMWBS) [16]	Mental well-being	Strengths
Patient Health Questionnaire 8 Items (PHQ-8) [75]	Depression	Deficits
Positive and Negative Affect Schedule (PANAS) [76]	Positive affect and negative affect	Deficits and strengths
Quick Inventory of Depressive Symptomatology 16 Items (QIDS-SR)^a^ [77]	Depression	Deficits
Alcohol Use Disorder Identification Test (AUDIT) [78]	Alcohol use	Deficits
Eating Disorder Examination Questionnaire (EDE-Q) [79]	Eating disorder	Deficits
Impact of Event Scale-Revised (IES-R) [80] (first edition) [273]	Posttraumatic stress disorder	Deficits
Medical Outcome Survey Short Form 36 Items (SF-36)^b^ [81,82,274]	Vitality, social functioning, role-emotional, and mental health	Deficits and strengths
Panic Disorder Severity Scale-Self Report 7 Items (PDSS-SR) [83,84]	Panic disorder	Deficits
Perceived Stress Scale 14 Items (PSS-14) [70,71]	Stress	Deficits
Perceived Stress Scale 4 Items (PSS-4) [71]	Stress	Deficits
WHOQOL-BREF^b,c^ [85]	Psychological (positive feelings, cognitions, self-esteem, body image, negative feelings, and spirituality)	Deficits and strengths
EQ-5D (also known as EQ-5D-3L)^b^ [86]	Anxiety or depression	Deficits
Mental Health Continuum Short-Form (MHC-SF) [14,15]	Mental well-being (emotional well-being, psychological well-being, and social well-being)	Strengths
Spielberger State-Trait Anxiety Inventory (STAI) [87]	Anxiety (state and trait)	Deficits

^a^These instruments were developed or psychometrically evaluated using item response theory during the first publication of the instruments.

^b^These are instruments measuring quality-of-life domains; only relevant mental health constructs are listed.

^c^WHOQOL-BREF: The World Health Organization Quality of Life (abbreviated version).

### Instrument Synthesis

Some instruments in Table 1 are shorter versions of the same instruments, which are listed separately due to usage differences. For example, Patient Health Questionnaire 8 Items [75] is identical to PHQ-9 [18] except without the item measuring suicidal thoughts or self-harm. Likewise, the Perceived Stress Scale 10 Items (PSS-10) and Perceived Stress Scale 4 Items are shorter versions of the Perceived Stress Scale 14 Items [70,71]. The 24 instruments that were included for synthesis in Table 1 can be loosely categorized into 3 groups: deficits-focused (18 instruments, 75%), strengths-focused (3 instruments, 12.5%), and “holistic” (deficit and strength, 3 instruments, 12.5%). The top 7 most commonly used instruments were deficits-focused, measuring perceived stress (eg, Perceived Stress Scale 14 Items), psychological distress (eg, K10), and early symptoms of anxiety (eg, Generalized Anxiety Disorder 7 Items [GAD-7]) and depression (eg, PHQ-9). In contrast, among the 3 strengths-focused instruments, the Satisfaction With Life Scale (SWL) measures life satisfaction while the other 2 (Warwick-Edinburgh Mental Well-Being Scale [WEMWBS] and Mental Health Continuum Short-Form [MHC-SF]) measure broad mental well-being constructs. SWL has the highest usage compared to the other 2 strengths-focused instruments. Among the 3 instruments measuring deficits- and strengths-based constructs, 2 measure health-related quality-of-life (HRQoL) instruments (Medical Outcome Survey Short Form 36 Items [SF-36] and The World Health Organization Quality of Life [abbreviated version, WHOQOL-BREF]), which typically subsume multiple constructs in different domains apart from mental health, such as physical health and functioning. EQ-5D is an HRQoL instrument, but was categorized as a deficits-focused instrument here because it measures only symptoms of anxiety or depression within the mental health domain. The final “holistic” measure was the Positive and Negative Affect Schedule, which measures positive and negative affect.

It is noteworthy that the original versions of all 24 instruments were developed and published before the year 2008 (Table 1 and RQ2 under Section S2 in Multimedia Appendix 3). Indeed, the Center for Epidemiologic Studies-Depression Scale (CES-D), PSS (14-item, 10-item, and 4-item), Hospital Anxiety and Depression Scale, Spielberger State-Trait Anxiety Inventory, SWL, and Positive and Negative Affect Schedule were published before the year 1990. Between the years 1990 and 2000, instruments measuring symptoms of mental illness other than anxiety and depression emerged such as Alcohol Use Disorder Identification Test (measuring alcohol use), Eating Disorder Examination Questionnaire (measuring symptoms of eating disorders), Impact of Event Scale-Revised (measuring symptoms of posttraumatic stress disorder) and Panic Disorder Severity Scale-Self Report 7 Items (measuring symptoms of panic disorder). During this period, SF-36, an HRQoL instrument, was also being developed. From the year 2000 onward, instruments screening symptoms of anxiety and depression, and measuring multifaceted mental well-being began to arise, such as PHQ-9, GAD-7, K10, MHC-SF, and WEMWBS. The HRQoL instrument WHOQOL-BREF was also developed after the year 2000.

Only 2 instruments (K10 and QIDS-SR) used IRT in the original publication (annotated ^a^ in Table 1 and RQ2 under Section S2 in Multimedia Appendix 3). The remaining instruments were typically developed using the factor analytical approach of CTT. It is noteworthy that the emerging PROMIS instruments, which were developed with digital administration in mind using contemporary test construction approaches such as IRT and CAT, have lower usage (less than 4/393, 1% of the total usage in Figure 4) compared to other instruments that were originally developed in paper-and-pen format.

### Instrument Usages

**Figure 5:**
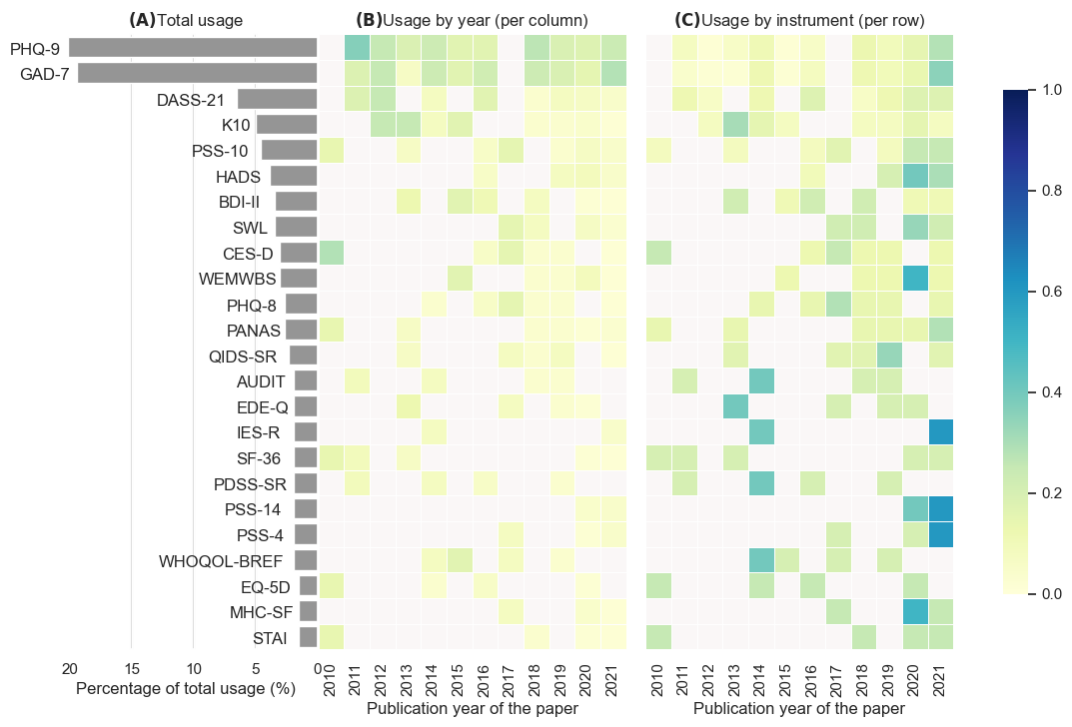
(A) Total usage of each instrument. (B) The usage percentage of each instrument per publication year of the record. (C) The usage percentage per instrument across years. AUDIT: Alcohol Use Disorder Identification Test; BDI-II: Beck Depression Inventory II; CES-D: Center for Epidemiologic Studies-Depression Scale; DASS-21: Depression, Anxiety, Stress Scale 21 Items; EDE-Q: Eating Disorder Examination Questionnaire; GAD-7: Generalized Anxiety Disorder 7 Items; HADS: Hospital Anxiety and Depression Scale; IES-R: Impact of Event Scale-Revised; K10: Kessler Psychological Distress Scale; MHC-SF: Mental Health Continuum Short-Form; PANAS: Positive and Negative Affect Schedule; PDSS-SR: Panic Disorder Severity Scale-Self Report 7 Items; PHQ-8: Patient Health Questionnaire 8 Items; PHQ-9: Patient Health Questionnaire 9 Items; PSS-4: Perceived Stress Scale 4 Items; PSS-10: Perceived Stress Scale 10 Items; PSS-14: Perceived Stress Scale 14 Items; QID-SR: Quick Inventory of Depressive Symptomatology 16 Items; SF-36: Medical Outcome Survey Short Form 36 Items; STAI: Spielberger State-Trait Anxiety Inventory; SWL: Satisfaction With Life Scale; WEMWBS: Warwick-Edinburgh Mental Well-Being Scale; WHOQOL-BREF: The World Health Organization Quality of Life (abbreviated version).

Figure 5 presents the 24 instruments’ usage from different perspectives across 147 studies. Figure 5A shows the distribution of the usage percentage of instruments. The top 3 instruments, which are deficits-focused, collectively contributed 46.2% (121/262) of the total usage of the 24 instruments (N=262): PHQ-9 (53/262, 20.2%), GAD-7 (51/262, 19.5%), and Depression, Anxiety, Stress Scale 21 Items (DASS-21; 17/262, 6.5%). Figure 5B breaks down the instrument’s usage by the record’s publication year. For each instrument, the usage pattern each year appeared to mimic its total usage pattern (Figure 5A), with the top 2 instruments (PHQ-9 and GAD-7) showing consistently high usage patterns across years since 2010. All included instruments were developed and released before 2010, so there is no need to consider the creation year when calculating usage frequency, as defined in our protocol [56]. Figure 5C presents a different perspective on each instrument. This figure shows the usage frequency by instrument (between 2010-2021), in which most instruments have higher usage toward the years 2020 and 2021, most likely influenced by the number of published records in 2020 and 2021 (Multimedia Appendix 5). Nonetheless, some top instruments (eg, PHQ-9, GAD-7, DASS-21, K10, and PSS-10) have been used almost consistently since 2010 among the included studies. In contrast, instruments measuring mental well-being, such as the SWL, WEMWBS, and MHC-SF, have only been used since 2015.

### Study Characteristics

Table 2 summarizes how the 24 instruments were administered in the studies identified (n=147). Among the included studies (grouped by instruments) in Table 2, each instrument was administered in various digital modalities and frequencies to the general population. The most common digital modalities for administering these instruments were through web applications accessible by any internet-connected device and mobile apps. The frequency of administration of the included instruments varies from multiple times a day [275,276] to yearly [277,278]. Studies that administered the same instrument multiple times per day typically used an experience sampling design such as ecological momentary assessment (EMA) [279]. The response time frame of instruments was typically adapted by including studies despite the original scales having a default recommended duration (eg, past 2 weeks and past 4 weeks).

**Table 2:** The attributes of the included studies are grouped by selected instruments.

Instrument and associated studies	Administration mode	Administration frequency	Response time frame	Study design	Country
PHQ-9^a^ [88,89,280-330]	Laptop or computer, mobile app, web application, or tablet or phone	1 day, 1 week, 12 months, 6 months, >1 month, >1 year, every 6 days, fortnightly, monthly, on-demand, or weekly	Past 2 weeks, past 4 weeks, past 6 days, or past week	EMA^b^, RCT^c^, cohort study, internet survey, or pre-post	UK, Ireland, USA, Canada, Australia, or New Zealand
GAD-7^d^ [88,89,280,282-286,288, 289,292,294,295,297,299-306,309, 311,313,317,318,320-327, 329-344]	Laptop or computer; mobile app; web application; SMS, text or email; or tablet or phone	1 day, 1 month, 1 week, 11 months, 3 weeks, >1 month, >1 year, every 6 days, fortnightly, monthly, on-demand, or weekly	Past 2 weeks, past 4 weeks, past 6 days, past 7 days	EMA, RCT, cohort study, internet survey, pre-post, or repeated cross-sectional	UK, Ireland, USA, Canada, Australia, New Zealand, or Singapore
DASS-21^e^ [90,283,324,345-358]	Mobile app and web application	1 month, 2 weeks, >1 month, >1 year, or weekly	Past 6 months or past week	EMA, RCT, case series, cohort study, or pre-post	Canada, Australia, USA, New Zealand, or UK
K10^f^ [88,89,277,278,289, 291,302,303,312,315,323, 330,359]	Web application	1 month, 1 week, 3 weeks, 4 times over 90 days, >1 month, 6 times over 10 weeks, or yearly	Past 2 weeks, past 28 days, past 30 days, past 4 weeks, or past month or last month	EMA, RCT, cohort study, internet survey, or pre-post	Canada, Australia, UK, Ireland, USA, New Zealand, or UK
PSS-10^g^ [277,311,343,351,360-367]	Mobile app and web application	2 weeks, 3 weeks, >1 month, or yearly	Past month or last month, or past week or last week	EMA, RCT, cohort study, internet survey, or pre-post	USA, Australia, UK, or Canada
HADS^h^ [312,368-376]	ACASI^i^, mobile app, or web application	1 month, 1 year, 6 months, >1 month, every 6 months, or on-demand	Past week or last week	RCT or cohort study	Australia, UK, USA, Singapore, or Canada
BDI-II^j^ [291,312,331,334,353,377-380]^k^	Web application	1 month or >1 month	Past 2 weeks	RCT, cohort study, or pre-post	Australia, Ireland, USA, or Hong Kong
SWL^l^ [90,91,275,288,357,365,381-383]	Mobile app or web application	1 month, 1 week, 30 days, >1 month, multiple times per day, on-demand, or same day	Now	EMA, RCT, cohort study, or pre-post	UK, Australia, New Zealand, or USA
CES-D^m^ [91,338,366,381,384-387]	Laptop or computer, or web application	1 month, 1 week, 3 weeks, 6 months, or >1 month	Past week or last week	RCT, cohort study, or repeated cross-sectional	Canada, USA, Australia, UK, or nonspecified countries from Asia and Europe
WEMWBS^n^ [276,278,295,306,373,388-390]	Mobile app or web application	1 month, 4 weeks, >1 month, multiple times per day, or yearly	Now or past 2 weeks	EMA, RCT, cohort study, or internet survey	UK or USA
PHQ-8^o^ [92,339,360,391-394]	Mobile app, web application, or tablet or phone	1 year, 2 weeks, 4 weeks, 6 months, >1 month, or on-demand	Past 2 weeks	EMA, RCT, cohort study, or pre-post	UK, USA, or Australia
PANAS^p^ [327,351,358,364,386,395,396]	Web application	1 week, 2 weeks, 4 weeks, 6 months, >1 month, or daily	General, moment, past few days, past few weeks, today, week, or year	EMA, RCT, cohort study, or pre-post	USA, Canada, UK, or Australia
QIDS-SR^q^ [93,333,397-400]	Mobile app; web application; SMS, text, or email; or tablet or phone	1 year, 2 weeks, >1 month, daily, or weekly	Past 7 days	EMA, RCT, or cohort study	Canada, UK, or USA
AUDIT^r^ [318,345,346,368,401]	ACASI or web application	1 year, >1 month, >1 year, or every 6 months	Past year or last year	RCT, cohort study, or pre-post	UK, Australia, or Canada
EDE-Q^s^ [277,347,378,402,403]	Web application	1 month, 6 months, >1 month, >1 year, or yearly	Past 28 days	RCT, cohort study, or pre-post	UK, Australia, Hong Kong, or USA
IES-R^t^ [292,301,374,404,405]	Web application	1 year, 6 months, or >1 month	Past 7 days	RCT, cohort study, or internet survey	Ireland, Australia, or USA
SF-36^u^ [357,363,378,395,406]	Web application	1 month, 2 weeks, >1 month, or biannually	Past 4 weeks	RCT, cohort study, or pre-post	Australia, New Zealand, USA, or Hong Kong
PDSS-SR^v^ [88,283,284,323,324]	Web application	6 months, >1 month, monthly, or weekly	Past week	RCT, cohort study, or pre-post	Canada or Australia
PSS-4^w^ [336,340,402,407,408]	Mobile app or web application	12 months, 6 months, >1 month, monthly, or weekly	Last 30 days, or past month or last month	RCT or cohort study	USA, Singapore, or UK
PSS-14^x^ [94,358,389,390,409]	Web application	1 month, >1 month, or daily	Past month or last month, or today	RCT, cohort study, or pre-post	USA, Canada, or UK
WHOQOL-BREF^y^ [286,318,324,397,410]	Web application	1 year, 2 weeks, or >1 month	Past 4 weeks	RCT or pre-post	UK, Canada, or USA
EQ-5D [313,322,411,412]	Web application	1 month, 12 months, or >1 month	Today	RCT or pre-post	Australia, UK, or Canada
MHC-SF^z^ [90,95,352,413]	Web application; or SMS, text, or email	1 month, >1 month, or multiple times per day	Since the last prompt or beep	EMA, RCT, or pre-post	Australia or USA
STAI^aa^ [340,363,380,414]	Web application	2 weeks, >1 month, or weekly	Now	Cohort study or pre-post	USA

^a^PHQ-9: Patient Health Questionnaire 9 Items.

^b^EMA: ecological momentary assessment.

^c^RCT: randomized controlled trial.

^d^GAD-7: Generalized Anxiety Disorder 7 Items.

^e^DASS-21: Depression, Anxiety, Stress Scale 21 Items.

^f^K10: Kessler Psychological Distress Scale.

^g^PSS-10: Perceived Stress Scale 10 Items.

^h^HADS: Hospital Anxiety and Depression Scale.

^i^ACASI: audio computer-assisted self-interview.

^j^BDI-II: Beck Depression Inventory II.

^k^This was administered only to the general public who were at risk or diagnosed with a physical or mental illness, not to the healthy general population.

^l^SWL: Satisfaction With Life Scale.

^m^CES-D: Center for Epidemiologic Studies-Depression Scale.

^n^WEMWBS: Warwick-Edinburgh Mental Well-Being Scale.

^o^PHQ-8: Patient Health Questionnaire 8 Items.

^p^PANAS: Positive and Negative Affect Schedule.

^q^QID-SR: Quick Inventory of Depressive Symptomatology 16 Items.

^r^AUDIT: Alcohol Use Disorder Identification Test.

^s^EDE-Q: Eating Disorder Examination Questionnaire.

^t^IES-R: Impact of Event Scale-Revised.

^u^SF-36: Medical Outcome Survey Short Form 36 Items.

^v^PDSS-SR: Panic Disorder Severity Scale-Self Report 7 Items.

^w^PSS-4: Perceived Stress Scale 4 Items.

^x^PSS-14: Perceived Stress Scale 14 Items.

^y^WHOQOL-BREF: The World Health Organization Quality of Life (abbreviated version).

^z^MHC-SF: Mental Health Continuum Short-Form.

^aa^STAI: Spielberger State-Trait Anxiety Inventory.

### Comparison With Existing Literature

The set of frequently used instruments identified here broadly aligns with findings of past reviews examining outcome measurements. Points of difference are likely due to the unique focus of this review on digital and longitudinal applications of the instruments. The commonly used measurement instruments that Breedvelt et al [66] found were included in the initial 68 instruments in the present review, except for the Edinburgh Postnatal Depression Scale [415] and General Health Questionnaire-12 [416]. The Edinburgh Postnatal Depression Scale was excluded because it is outside the scope of the present review. It is noteworthy that Breedvelt et al [66] found that the CES-D was the most commonly used instrument in measuring depression, followed by the Beck Depression Inventory II and the PHQ-9, whereas in the present review, we found that the usage of the PHQ-9 is substantially higher than that of any other instrument. This difference may be due to the present focus on digital, repeated measures application, especially given that the PHQ-9 is commonly used in mobile apps for EMA studies [417,418].

Most instruments measuring mental well-being and HRQoL found in the present review were also identified in an earlier review by Lindert et al [61] of well-being measurement scales. An exception, however, is the MHC-SF. The difference in review timeframes between Lindert et al (2007-2012) [61] and the present review (2010-2021) may explain this discrepancy. Another plausible explanation is that Lindert et al [61] focused on subjective well-being, whereas MHC-SF is conceptually broader than subjective well-being. In contrast, a recent scoping review focusing on the dual-continua model of mental health [55] found SWL and MHC-SF to be the most used measurements for positive mental health—both instruments appear in Table 1 here. Beidas et al [96] found 29 validated mental health screening instruments for the general adult population that are free and brief. Some instruments found in the present review, such as the DASS-21, CES-D, K10, and PSS-10, were not included in their review, most likely due to differences in search scopes and review aims.

## Discussion

### Overview

The present scoping review aimed to advance our understanding of the range of instruments that could function as mental health self-monitoring tools in the digital era. This was achieved by identifying empirical studies that administered instruments in (1) the general population, (2) digital format, and (3) longitudinal designs. To the best of our knowledge, this is the first review that focuses on studies that administered instruments meeting these 3 criteria. The primary outcome was an ordered list of commonly used instruments that measure mental health longitudinally using digital technologies in the general population.

### Principal Findings

The literature review and synthesis generated 2 major findings. First, among the 24 most commonly used instruments, the majority (18/24, 75%) were deficits-focused. PHQ-9 and GAD-7 were by far the most used instruments, with consistent yearly usage between the years 2010 and 2021, measuring symptoms of depression and generalized anxiety, correspondingly. In the present review, the considerably higher usage of PHQ-9 and GAD-7 (Figure 5) was unsurprising because they have been used extensively as longitudinal measures for mental health, from web-based applications to mobile apps for EMA studies [417,418]. These 2 instruments are also nominated for use in a recent global effort to standardize mental health measures [419].

The list of commonly used instruments also included strengths-focused instruments (eg, SWL, WEMWBS, and MHC-SF). Instruments measuring HRQoL (eg, SF-36 and WHOQOL-BREF) are a different type of commonly used instrument—from Keyes’ framework, they can be understood as measuring aspects of deficits-based (eg, anxiety or depression) and strengths-based (eg, positive effects or energy level) mental health constructs in conjunction with ratings of physical health domains and functioning. Among the strengths-focused instruments, SWL, WEMWBS, and MHC-SF, measuring life satisfaction and mental well-being, were found to be the most used instruments.

The predominance of deficits-focused instruments found here is at least partly due to the traditional elevation of illness-focused constructs in psychology and mental health research and practice (not to mention medicine and psychiatry [420]). The granularity of the deficits-based constructs measured by these instruments may also contribute to their disproportionate usage. For example, deficits-focused instruments are typically unidimensional and high-fidelity [421,422] as these instruments emphasize the accuracy and specificity of screening for or diagnosing specific mental disorders. In contrast, strengths-focused instruments and HRQoL instruments are typically multidimensional, containing broad constructs of measurement (also known as high bandwidth), addressing the complexity and variability of the domain of mental health or general health [62,423].

It is important to note that, used in isolation, the population deficits-focused instruments identified here may have limited utility as longitudinal mental health self-monitoring tools in the general population. The prevalence of mental disorders in the general population is relatively low, so deficits-focused instruments measuring symptoms of mental illnesses tend to exhibit floor effects [97,424,425]. While these screening instruments play a role in potentially detecting early symptoms of mental illnesses, they may not be so helpful in providing a general picture of individuals’ mental health over time. The dual continua model of mental health suggests that measuring strengths-based constructs could complement deficits-focused measurement and consequently provide more ecologically valid and statistically sensitive assessments for the general population.

Second, while the 24 instruments synthesized in this review have been frequently administered in digital modalities (1 of the review’s 3 inclusion criteria), they were all developed at least 2 decades ago, and most followed CTT and were designed for paper-and-pen format. Since then, we have seen substantial work using IRT to revalidate the psychometric properties of these instruments [426-428], but (except for the PROMIS project) less attention has been devoted to new instrument development.

Next-generation mental health assessments could harness the computational power offered by digital technologies, coupled with contemporary psychometric practices such as IRT and computerized adaptive testing, to advance evidence-based assessments. Undoubtedly, a holistic instrument that measures both deficits- and strengths-based constructs may contain a larger number of self-report items compared to deficits- or strengths-based instruments alone. However, the combination of IRT and computerized adaptive testing can significantly reduce the number of questions asked in a survey [35], which reduces respondent fatigue burden [429], a common phenomenon where participants lose motivation when completing long or repeated surveys, affecting the data quality. This phenomenon is particularly prominent in self-monitoring instruments, which are typically administered multiple times over a period [430]. Therefore, there is a golden opportunity to lift the capability of evidence-based assessments through digital technologies, which provide an excellent launching platform for a digital tool that encourages people to monitor their mental health.

### Limitations

Due to the sheer volume of eligible records for full-text screening, for pragmatic reasons, we randomly selected 4 batches of 250 records (a total of 1000 records) of the 8460 eligible records for full-text screening and synthesis after the preliminary screening. Although we demonstrated that the distributions of instruments’ usages were statistically indicative of the full records, we cannot definitively exclude the possibility that this pragmatic strategy biased our findings. However, given the prominence of a few commonly used instruments at the top of the list (eg, PHQ-9 and GAD-7) and previous studies in the literature, it seems unlikely that we missed any instruments that would have warranted inclusion in the top 30 (Figure 4). Our primary objective is to develop a novel ranking of the most frequently used instruments that are potentially suitable for mental health self-monitoring in the general population. Future studies can elaborate on and update the results presented in the present study by considering records that were not selected for full-text screening. Additionally, the restriction on English-only instruments in this review may limit the generalizability of the results.

### Implications

This study has several implications for future research. First, the finding that deficits-focused instruments predominate the ranking of usage frequency despite their known distributional problems in the general population (floor effects) calls for serious consideration of more holistic instruments. As positive psychology, recovery, and mental health promotion and prevention gain traction [420], future research studies should work toward the development of more holistic measures, potentially informed by the dual continua model [431-434]. Such measures for general population use would adequately capture both deficits (constructs relevant to a significant minority of the population, and of great interest clinically) and strengths (constructs that have the potential to support the majority of the population to advance their positive mental health).

Second, the 24 instruments included in the final synthesis of measures, while commonly used, were originally designed more than a decade ago for use in paper-and-pen format without taking advantage of the great possibilities of digital measurement, such as computerized adaptive testing. Although these instruments have been translated into digital format, we observed that the implications of such direct translation may not have been fully investigated, especially when these instruments were administered in repeated measurements with short intervals (eg, multiple times per day), such as EMA studies.

Finally, we highlight that advances in technology’s power and reach (eg, smartphones [435-437]) and developments in psychometric practice (eg, IRT and computerized adaptive testing [34,35]) provide exciting opportunities for optimizing digital delivery of psychological measures. While existing deficits-focused instruments could be (and often are) used alongside a strengths-focused measure (eg, HRQoL [438] or SWL [439]), this strategy has some significant limitations. Specifically, it does not recognize the overlap between the 2 dimensions of mental health as proposed in the dual continua model, generates unnecessary response burden, and limits construct validity. Given that the intercorrelated relationship between deficits- and strengths-based mental health constructs is well-established in the literature [14,55], measuring both of them in a single instrument, as we argued, has the optimal construct validity. Theoretically, with the advances in digital technologies, a next-generation low-burden mental health monitoring tool that can measure both deficits and strengths of individuals’ mental health that is relevant and engaging is entirely feasible. Indeed, findings from the present review suggest that a novel computerized adaptive testing instrument could be purposely built to achieve this aim [440].

### Conclusions

Although the last decade has seen a shift in the conceptualization of mental health to recognize strengths-focused concepts alongside traditional deficits-focused constructs, our review found that when it comes to an important translational opportunity—measuring mental health digitally and longitudinally in the general population, as is required for mental health monitoring, the literature remains skewed toward illness- and deficits-based constructs. The review also found that this legacy conceptualization of mental health comes along with a legacy approach to test development. We conclude that the computational power and ubiquity of digital technologies have untapped potential to advance mental health measurement by supporting a more holistic characterization of the complexity intrinsic to the mental health construct. Specifically, IRT-based development of a dual-continua mental health instrument to be delivered via CAT is an exciting possibility for the near future.

## Appendix

Multimedia Appendix 1 The conceptualization of mental health in this review. This diagram depicts our conceptualization of mental health to guide our search strategy and formulation of search terms. A copy of this figure is also available in our protocol [1].

Multimedia Appendix 2 Mapping of the research questions between the protocol and the present scoping review.

Multimedia Appendix 3 Search protocols and ancillary information on instruments.

Multimedia Appendix 4 The decision flowchart to determine if an instrument used in a paper is in English.

Multimedia Appendix 5 The distribution of the publication years of the included studies.

Multimedia Appendix 6 PRISMA 2020 Checklist.

## Abbreviations

CAT: Computerized Adaptive Test
CES-D: Center for Epidemiologic Studies-Depression Scale
COSMIN: COnsensus-based Standards for the selection of health Measurement INstruments
CTT: classical test theory
DASS-21: Depression, Anxiety, Stress Scale 21 Items
EMA: ecological momentary assessment
GAD-7: Generalized Anxiety Disorder 7 Items
HRQoL: health-related quality-of-life
IRT: item response theory
K10: Kessler Psychological Distress Scale
MHC-SF: Mental Health Continuum Short-Form
PHQ-8: Patient Health Questionnaire 8 Items
PHQ-9: Patient Health Questionnaire 9 Items
PICOT: Population, intervention, comparison, outcome, and time
PRISMA-ScR: Preferred Reporting Items for Systematic Review and Meta-analysis Extension for Scoping Reviews
PROMIS: Patient Reported Outcome Measures Information System
PSS-10: Perceived Stress Scale 10 Items
RQ: research question
SF-36: Medical Outcome Survey Short Form 36 Items
SWL: Satisfaction With Life Scale
WEMWBS: Warwick-Edinburgh Mental Well-Being Scale
WHOQOL-BREF: The World Health Organization Quality of Life (abbreviated version)

## Data Availability

Data, code, and other materials generated or analyzed during this review are available from the corresponding author on reasonable request.
