# Supplementary figures and images for: Toward Digital Self-Monitoring of Mental Health in the General Population: Scoping Review of Existing Approaches to Self-Report Measurement
Source: JMIR Ment Health. 2025 Sep 18;12:e59351. doi: 10.2196/59351 (PMC12491901; doi:10.2196/59351)

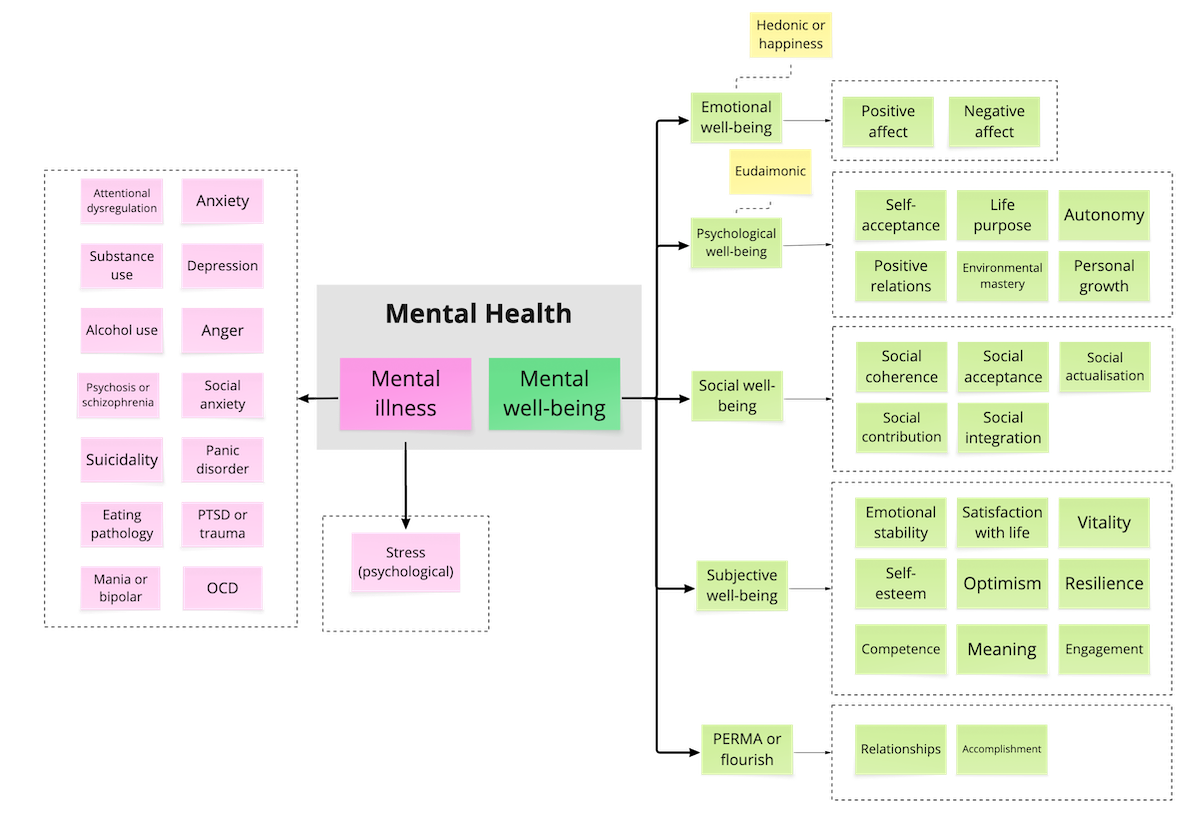

Supplement: Multimedia Appendix 1 [file mental_v12i1e59351_app1.png]

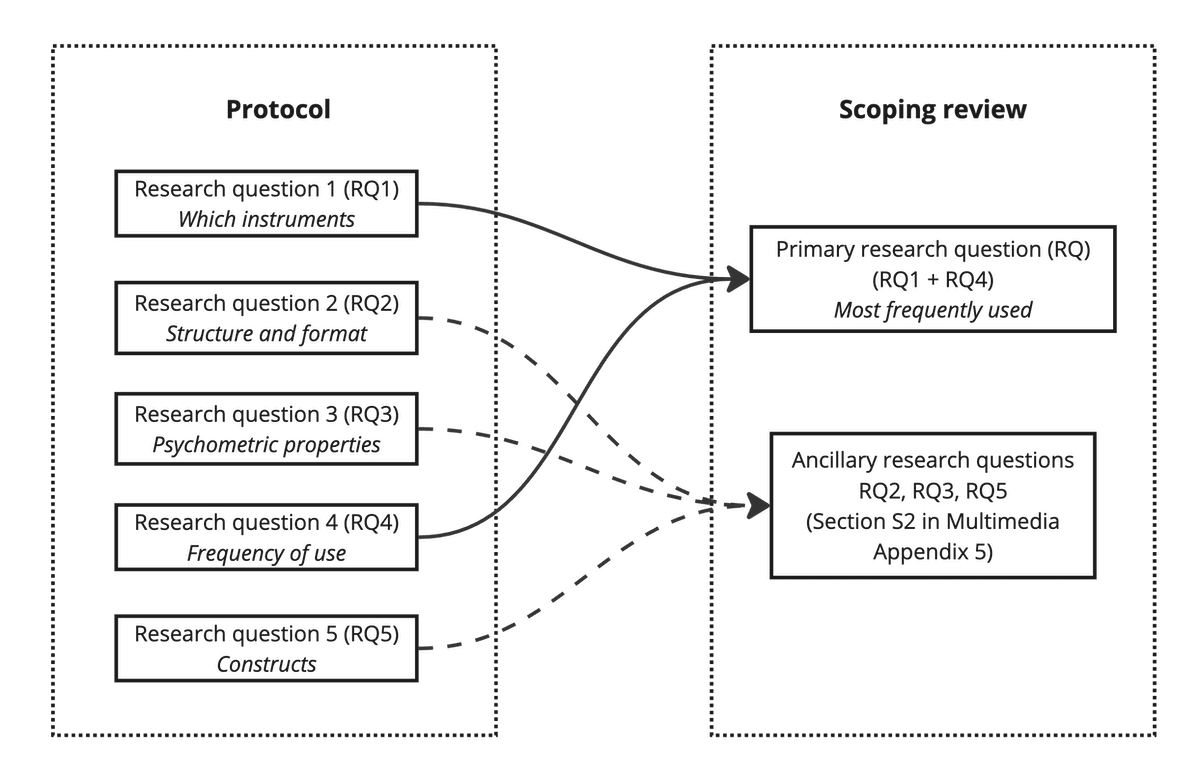

Supplement: Multimedia Appendix 2 [file mental_v12i1e59351_app2.png]

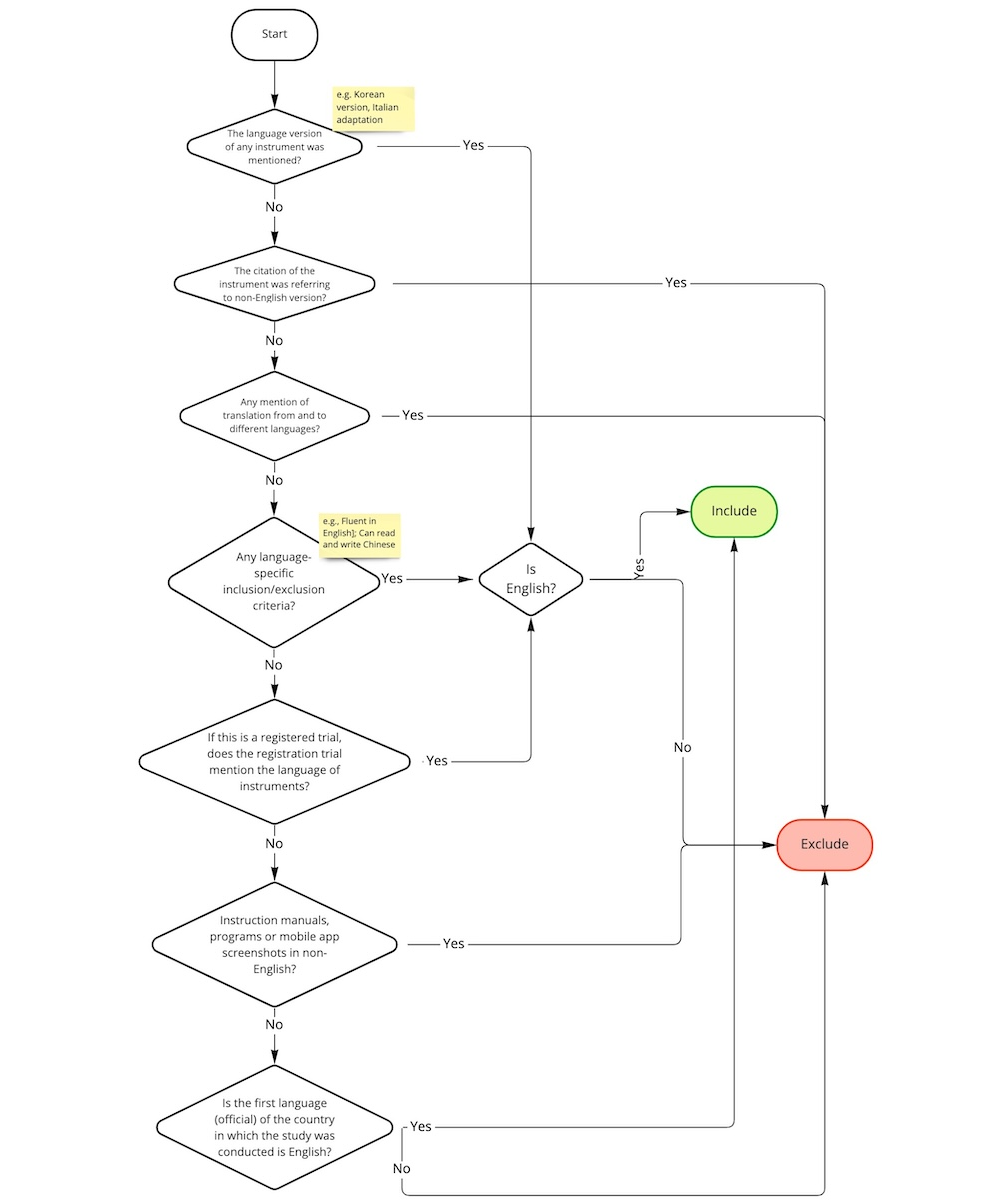

Supplement: Multimedia Appendix 4 [file mental_v12i1e59351_app4.png]

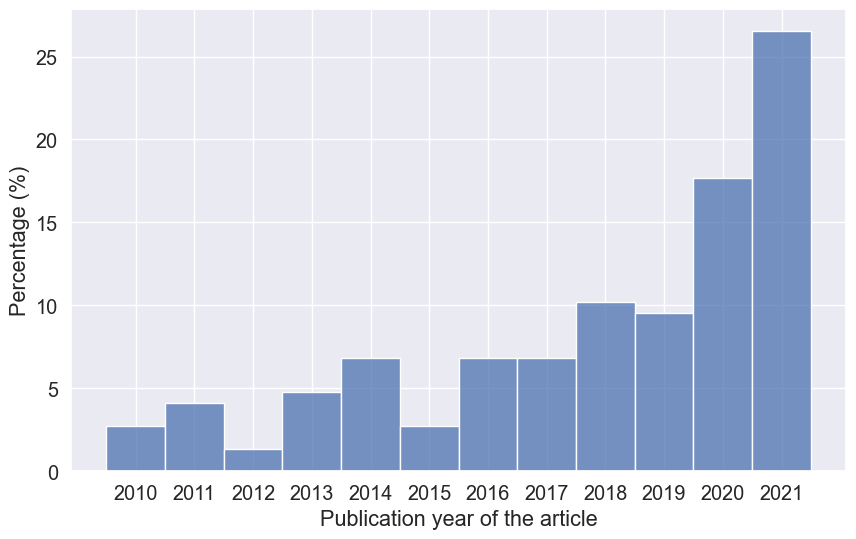

Supplement: Multimedia Appendix 5 [file mental_v12i1e59351_app5.png]
