# Supplementary material for: Toward Digital Self-Monitoring of Mental Health in the General Population: Scoping Review of Existing Approaches to Self-Report Measurement
Source: JMIR Ment Health. 2025 Sep 18;12:e59351. doi: 10.2196/59351 (PMC12491901; doi:10.2196/59351)
Supplement: Multimedia Appendix 3 [file mental_v12i1e59351_app3.docx]

## Section S1 - Search Protocols

The search strategy was developed based on the eligibility criteria defined in our protocol [[1](#ref-Koh2023-ok)] and peer-reviewed by a librarian from Swinburne University of Technology who has expertise in developing searches for systematic reviews. The search strategy consists of four high-level concepts: mental health, instruments, repeated measures and digital/online (see Table S1). The search terms targeted the title, abstract and keywords and combined with Boolean operators.

The following table explains the four concepts that guided our search strategies in this review before we included full search queries for each database that we queried.

**Table S1** *The four search concepts that guide our search strategy.*

| Mental Health | We adopted a broad definition of mental health, which includes strengths-based constructs (e.g., mental well-being) and deficit-based constructs (e.g., mental illnesses). The search terms for mental disorders are adapted from the review conducted by Beidas and colleagues [[2](#ref-Beidas2015-ht)]. Please refer to the full search queries below for these search terms. |
| --- | --- |
| Instruments | assessment* OR index* OR indicator* OR indices OR instrument* OR inventor* OR measure* OR questionnair* OR scale* OR subscale* OR survey* OR tool* |
| Multiple measures across time | (“pre* post*” OR ”pre-post”) OR (”random*” AND “trial*”) OR baseline OR ”cohort stud*” OR “experience sampling” OR “follow up” OR “long term” OR longitudin* OR monitor* OR nation* OR population OR prospective OR “public health” OR “quality of life” OR repeat* OR routine OR screen* OR surveillance* OR “time point*” OR timepoint* OR wave* |
| Digital/online | app OR apps OR “cell* phone” OR computer OR “digital health” OR ehealth OR “electronic health” OR “health kiosk*” OR ”health station*” OR internet OR mhealth OR “mobile device*” OR “mobile health” OR “mobile phone” OR online OR “smart phone” OR smartphone OR web* |

For the concept of multiple measures across time, a potential secondary application of the new tool would be to measure group-level change in a population across time.

Two filters were used:

- Publication Year between 01 Jan 2010 - 31 Dec 2021 (inclusive)
- Language = English

## Full search queries for each database

### Scopus

TITLE-ABS-KEY(flourish* OR languish* OR {mental health} OR {mental disorder} OR {mental illness} OR agoraphobia OR panic OR {post-traumatic} OR posttraumatic OR ptsd OR trauma OR {obsessive-compulsive disorder} OR ocd OR adhd OR {attention deficit disorder} OR {attention deficit hyperactivity disorder} OR {substance use} OR psychosis OR schizophrenia OR distress OR hopelessness OR {self-harm} OR "self-injur*" OR suicid* OR anxiety OR depression OR depressive OR anger OR {alcohol use} OR {anorexia nervosa} OR {bulimia nervosa} OR {eating disorder} OR {bipolar disorder} OR hypomani* OR mania OR stress OR {well-being} OR wellbeing OR {mental wellness} OR {emotional functioning} OR {emotional wellness} OR hedonic OR {affective valence} OR {negative affect} OR {negative emotion} OR {negative feeling} OR {positive affect} OR {positive emotion} OR {positive feeling} OR eudaimon* OR {psychological functioning} OR {psychological wellness} OR {hierarchy of needs} OR maslow OR {self-acceptance} OR {self-actualisation} OR {self-actualization} OR {life meaning} OR {life purpose} OR {meaning in life} OR {meaning to life} OR {purpose in life} OR autonomy OR individuat* OR {self-determination} OR "interpersonal relation*" OR "meaningful relation*" OR "social relation*" OR "environment* mastery" OR {sense of mastery} OR {personal growth} OR {social functioning} OR {social coherence} OR {social interest} OR {social acceptance} OR {social actualisation} OR {social actualization} OR {social growth} OR {community contribution} OR {social contribution} OR "social responsibilit*" OR {society contribution} OR {sense of belonging} OR {social belonging} OR {social cohesion} OR {social integration} OR {social isolation} OR {emotional stability} OR happiness OR {life satisfaction} OR {satisfaction with life} OR vitality OR {self-esteem} OR optimism OR resilience OR {sense of accomplishment} OR {self-worth} OR {life engagement} OR {psychological engagement} OR {social connection} OR {social relationship} OR {goal achievement} OR {self-efficacy}) AND TITLE-ABS-KEY(assessment* OR index* OR indicator* OR indices OR instrument* OR inventor* OR measure* OR questionnair* OR scale* OR subscale* OR survey* OR tool*) AND TITLE-ABS-KEY(("pre* post*" OR "pre-post") OR ("random*" AND "trial*") OR baseline OR "cohort stud*" OR {experience sampling} OR {follow up} OR {long term} OR longitudin* OR monitor* OR nation* OR population OR prospective OR {public health} OR {quality of life} OR repeat* OR routine OR screen* OR surveillance* OR "time point*" OR timepoint* OR wave*) AND TITLE-ABS-KEY(app OR apps OR "cell* phone" OR computer OR {digital health} OR ehealth OR {electronic health} OR "health kiosk*" OR "health station*" OR internet OR mhealth OR "mobile device*" OR {mobile health} OR {mobile phone} OR online OR {smart phone} OR smartphone OR web*) AND PUBYEAR > 2009 AND PUBYEAR < 2022 AND LANGUAGE ( "English" )

### Web of Science

TS=(flourish* OR languish* OR "mental health" OR "mental disorder" OR "mental illness" OR agoraphobia OR panic OR "post-traumatic" OR posttraumatic OR ptsd OR trauma OR "obsessive-compulsive disorder" OR ocd OR adhd OR "attention deficit disorder" OR "attention deficit hyperactivity disorder" OR "substance use" OR psychosis OR schizophrenia OR distress OR hopelessness OR "self-harm" OR "self-injur*" OR suicid* OR anxiety OR depression OR depressive OR anger OR "alcohol use" OR "anorexia nervosa" OR "bulimia nervosa" OR "eating disorder" OR "bipolar disorder" OR hypomani* OR mania OR stress OR "well-being" OR wellbeing OR "mental wellness" OR "emotional functioning" OR "emotional wellness" OR hedonic OR "affective valence" OR "negative affect" OR "negative emotion" OR "negative feeling" OR "positive affect" OR "positive emotion" OR "positive feeling" OR eudaimon* OR "psychological functioning" OR "psychological wellness" OR "hierarchy of needs" OR maslow OR "self-acceptance" OR "self-actualisation" OR "self-actualization" OR "life meaning" OR "life purpose" OR "meaning in life" OR "meaning to life" OR "purpose in life" OR autonomy OR individuat* OR "self-determination" OR "interpersonal relation*" OR "meaningful relation*" OR "social relation*" OR "environment* mastery" OR "sense of mastery" OR "personal growth" OR "social functioning" OR "social coherence" OR "social interest" OR "social acceptance" OR "social actualisation" OR "social actualization" OR "social growth" OR "community contribution" OR "social contribution" OR "social responsibilit*" OR "society contribution" OR "sense of belonging" OR "social belonging" OR "social cohesion" OR "social integration" OR "social isolation" OR "emotional stability" OR happiness OR "life satisfaction" OR "satisfaction with life" OR vitality OR "self-esteem" OR optimism OR resilience OR "sense of accomplishment" OR "self-worth" OR "life engagement" OR "psychological engagement" OR "social connection" OR "social relationship" OR "goal achievement" OR "self-efficacy")

 AND TS=(assessment* OR index* OR indicator* OR indices OR instrument* OR inventor* OR measure* OR questionnair* OR scale* OR subscale* OR survey* OR tool*)

 AND TS=(("pre* post*" OR "pre-post") OR ("random*" AND "trial*") OR baseline OR "cohort stud*" OR "experience sampling" OR "follow up" OR "long term" OR longitudin* OR monitor* OR nation* OR population OR prospective OR "public health" OR "quality of life" OR repeat* OR routine OR screen* OR surveillance* OR "time point*" OR timepoint* OR wave*)

 AND TS=(app OR apps OR "cell* phone" OR computer OR "digital health" OR ehealth OR "electronic health" OR "health kiosk*" OR "health station*" OR internet OR mhealth OR "mobile device*" OR "mobile health" OR "mobile phone" OR online OR "smart phone" OR smartphone OR web*)

 AND PY=(2010-2021)

### PubMed

("flourish*"[tw] OR "languish*"[tw] OR "mental health"[tw] OR "mental disorder"[tw] OR "mental illness"[tw] OR "agoraphobia"[tw] OR "panic"[tw] OR "post-traumatic"[tw] OR "posttraumatic"[tw] OR "ptsd"[tw] OR "trauma"[tw] OR "obsessive-compulsive disorder"[tw] OR "ocd"[tw] OR "adhd"[tw] OR "attention deficit disorder"[tw] OR "attention deficit hyperactivity disorder"[tw] OR "substance use"[tw] OR "psychosis"[tw] OR "schizophrenia"[tw] OR "distress"[tw] OR "hopelessness"[tw] OR "self-harm"[tw] OR "self-injur*"[tw] OR "suicid*"[tw] OR "anxiety"[tw] OR "depression"[tw] OR "depressive"[tw] OR "anger"[tw] OR "alcohol use"[tw] OR "anorexia nervosa"[tw] OR "bulimia nervosa"[tw] OR "eating disorder"[tw] OR "bipolar disorder"[tw] OR "hypomani*"[tw] OR "mania"[tw] OR "stress"[tw] OR "well-being"[tw] OR "wellbeing"[tw] OR "mental wellness"[tw] OR "emotional functioning"[tw] OR "emotional wellness"[tw] OR "hedonic"[tw] OR "affective valence"[tw] OR "negative affect"[tw] OR "negative emotion"[tw] OR "negative feeling"[tw] OR "positive affect"[tw] OR "positive emotion"[tw] OR "positive feeling"[tw] OR "eudaimon*"[tw] OR "psychological functioning"[tw] OR "psychological wellness"[tw] OR "hierarchy of needs"[tw] OR "maslow"[tw] OR "self-acceptance"[tw] OR "self-actualisation"[tw] OR "self-actualization"[tw] OR "life meaning"[tw] OR "life purpose"[tw] OR "meaning in life"[tw] OR "meaning to life"[tw] OR "purpose in life"[tw] OR "autonomy"[tw] OR "individuat*"[tw] OR "self-determination"[tw] OR "interpersonal relation*"[tw] OR "meaningful relation*"[tw] OR "social relation*"[tw] OR "environment* mastery"[tw] OR "sense of mastery"[tw] OR "personal growth"[tw] OR "social functioning"[tw] OR "social coherence"[tw] OR "social interest"[tw] OR "social acceptance"[tw] OR "social actualisation"[tw] OR "social actualization"[tw] OR "social growth"[tw] OR "community contribution"[tw] OR "social contribution"[tw] OR "social responsibilit*"[tw] OR "society contribution"[tw] OR "sense of belonging"[tw] OR "social belonging"[tw] OR "social cohesion"[tw] OR "social integration"[tw] OR "social isolation"[tw] OR "emotional stability"[tw] OR "happiness"[tw] OR "life satisfaction"[tw] OR "satisfaction with life"[tw] OR "vitality"[tw] OR "self-esteem"[tw] OR "optimism"[tw] OR "resilience"[tw] OR "sense of accomplishment"[tw] OR "self-worth"[tw] OR "life engagement"[tw] OR "psychological engagement"[tw] OR "social connection"[tw] OR "social relationship"[tw] OR "goal achievement"[tw] OR "self-efficacy"[tw]) AND ("assessment*"[tw] OR "index*"[tw] OR "indicator*"[tw] OR "indices"[tw] OR "instrument*"[tw] OR "inventor*"[tw] OR "measure*"[tw] OR "questionnair*"[tw] OR "scale*"[tw] OR "subscale*"[tw] OR "survey*"[tw] OR "tool*"[tw]) AND (("pre* post*" OR "pre-post") OR ("random*" AND "trial*") OR "baseline"[tw] OR "cohort stud*"[tw] OR "experience sampling"[tw] OR "follow up"[tw] OR "long term"[tw] OR "longitudin*"[tw] OR "monitor*"[tw] OR "nation*"[tw] OR "population"[tw] OR "prospective"[tw] OR "public health"[tw] OR "quality of life"[tw] OR "repeat*"[tw] OR "routine"[tw] OR "screen*"[tw] OR "surveillance*"[tw] OR "time point*"[tw] OR "timepoint*"[tw] OR "wave*"[tw]) AND ("app"[tw] OR "apps"[tw] OR "cell* phone"[tw] OR "computer"[tw] OR "digital health"[tw] OR "ehealth"[tw] OR "electronic health"[tw] OR "health kiosk*"[tw] OR "health station*"[tw] OR "internet"[tw] OR "mhealth"[tw] OR "mobile device*"[tw] OR "mobile health"[tw] OR "mobile phone"[tw] OR "online"[tw] OR "smart phone"[tw] OR "smartphone"[tw] OR "web*"[tw]) AND (("2010/01/01"[Date - Publication] : "2021/12/31"[Date - Publication]) AND (English[Language]))

### PsychInfo (via Ovid)

(flourish*.mp OR languish*.mp OR "mental health".mp OR "mental disorder".mp OR "mental illness".mp OR agoraphobia.mp OR panic.mp OR "post-traumatic".mp OR posttraumatic.mp OR ptsd.mp OR trauma.mp OR "obsessive-compulsive disorder".mp OR ocd.mp OR adhd.mp OR "attention deficit disorder".mp OR "attention deficit hyperactivity disorder".mp OR "substance use".mp OR psychosis.mp OR schizophrenia.mp OR distress.mp OR hopelessness.mp OR "self-harm".mp OR "self-injur*".mp OR suicid*.mp OR anxiety.mp OR depression.mp OR depressive.mp OR anger.mp OR "alcohol use".mp OR "anorexia nervosa".mp OR "bulimia nervosa".mp OR "eating disorder".mp OR "bipolar disorder".mp OR hypomani*.mp OR mania.mp OR stress.mp OR "well-being".mp OR wellbeing.mp OR "mental wellness".mp OR "emotional functioning".mp OR "emotional wellness".mp OR hedonic.mp OR "affective valence".mp OR "negative affect".mp OR "negative emotion".mp OR "negative feeling".mp OR "positive affect".mp OR "positive emotion".mp OR "positive feeling".mp OR eudaimon*.mp OR "psychological functioning".mp OR "psychological wellness".mp OR "hierarchy of needs".mp OR maslow.mp OR "self-acceptance".mp OR "self-actualisation".mp OR "self-actualization".mp OR "life meaning".mp OR "life purpose".mp OR "meaning in life".mp OR "meaning to life".mp OR "purpose in life".mp OR autonomy.mp OR individuat*.mp OR "self-determination".mp OR "interpersonal relation*".mp OR "meaningful relation*".mp OR "social relation*".mp OR "environment* mastery".mp OR "sense of mastery".mp OR "personal growth".mp OR "social functioning".mp OR "social coherence".mp OR "social interest".mp OR "social acceptance".mp OR "social actualisation".mp OR "social actualization".mp OR "social growth".mp OR "community contribution".mp OR "social contribution".mp OR "social responsibilit*".mp OR "society contribution".mp OR "sense of belonging".mp OR "social belonging".mp OR "social cohesion".mp OR "social integration".mp OR "social isolation".mp OR "emotional stability".mp OR happiness.mp OR "life satisfaction".mp OR "satisfaction with life".mp OR vitality.mp OR "self-esteem".mp OR optimism.mp OR resilience.mp OR "sense of accomplishment".mp OR "self-worth".mp OR "life engagement".mp OR "psychological engagement".mp OR "social connection".mp OR "social relationship".mp OR "goal achievement".mp OR "self-efficacy".mp)

 AND (assessment*.mp OR index*.mp OR indicator*.mp OR indices.mp OR instrument*.mp OR inventor*.mp OR measure*.mp OR questionnair*.mp OR scale*.mp OR subscale*.mp OR survey*.mp OR tool*.mp)

 AND (("pre* post*" OR "pre-post").mp OR ("random*" AND "trial*").mp OR baseline.mp OR "cohort stud*".mp OR "experience sampling".mp OR "follow up".mp OR "long term".mp OR longitudin*.mp OR monitor*.mp OR nation*.mp OR population.mp OR prospective.mp OR "public health".mp OR "quality of life".mp OR repeat*.mp OR routine.mp OR screen*.mp OR surveillance*.mp OR "time point*".mp OR timepoint*.mp OR wave*.mp)

 AND (app.mp OR apps.mp OR "cell* phone".mp OR computer.mp OR "digital health".mp OR ehealth.mp OR "electronic health".mp OR "health kiosk*".mp OR "health station*".mp OR internet.mp OR mhealth.mp OR "mobile device*".mp OR "mobile health".mp OR "mobile phone".mp OR online.mp OR "smart phone".mp OR smartphone.mp OR web*.mp)

with ‘Publication Year’ filter set to between 2010 and 2021 and Languages set to ‘English’.

### Psychology & Behavioral Sciences collection (via EBSCOhost)

(flourish* OR languish* OR "mental health" OR "mental disorder" OR "mental illness" OR agoraphobia OR panic OR "post-traumatic" OR posttraumatic OR ptsd OR trauma OR "obsessive-compulsive disorder" OR ocd OR adhd OR "attention deficit disorder" OR "attention deficit hyperactivity disorder" OR "substance use" OR psychosis OR schizophrenia OR distress OR hopelessness OR "self-harm" OR "self-injur*" OR suicid* OR anxiety OR depression OR depressive OR anger OR "alcohol use" OR "anorexia nervosa" OR "bulimia nervosa" OR "eating disorder" OR "bipolar disorder" OR hypomani* OR mania OR stress OR "well-being" OR wellbeing OR "mental wellness" OR "emotional functioning" OR "emotional wellness" OR hedonic OR "affective valence" OR "negative affect" OR "negative emotion" OR "negative feeling" OR "positive affect" OR "positive emotion" OR "positive feeling" OR eudaimon* OR "psychological functioning" OR "psychological wellness" OR "hierarchy of needs" OR maslow OR "self-acceptance" OR "self-actualisation" OR "self-actualization" OR "life meaning" OR "life purpose" OR "meaning in life" OR "meaning to life" OR "purpose in life" OR autonomy OR individuat* OR "self-determination" OR "interpersonal relation*" OR "meaningful relation*" OR "social relation*" OR "environment* mastery" OR "sense of mastery" OR "personal growth" OR "social functioning" OR "social coherence" OR "social interest" OR "social acceptance" OR "social actualisation" OR "social actualization" OR "social growth" OR "community contribution" OR "social contribution" OR "social responsibilit*" OR "society contribution" OR "sense of belonging" OR "social belonging" OR "social cohesion" OR "social integration" OR "social isolation" OR "emotional stability" OR happiness OR "life satisfaction" OR "satisfaction with life" OR vitality OR "self-esteem" OR optimism OR resilience OR "sense of accomplishment" OR "self-worth" OR "life engagement" OR "psychological engagement" OR "social connection" OR "social relationship" OR "goal achievement" OR "self-efficacy”)

AND (assessment* OR index* OR indicator* OR indices OR instrument* OR inventor* OR measure* OR questionnair* OR scale* OR subscale* OR survey* OR tool*)

AND (("pre* post*" OR "pre-post") OR ("random*" AND "trial*") OR baseline OR "cohort stud*" OR "experience sampling" OR "follow up" OR "long term" OR longitudin* OR monitor* OR nation* OR population OR prospective OR "public health" OR "quality of life" OR repeat* OR routine OR screen* OR surveillance* OR "time point*" OR timepoint* OR wave*)

AND (app OR apps OR "cell* phone" OR computer OR "digital health" OR ehealth OR "electronic health" OR "health kiosk*" OR "health station*" OR internet OR mhealth OR "mobile device*" OR "mobile health" OR "mobile phone" OR online OR "smart phone" OR smartphone OR web*)

with ‘Published Date’ filter set to ‘20100101-20211231’

## Section S2 - Ancillary Information on Instruments

For clarity of exposition, we reorganized the original five research questions defined in our protocol [[1](#ref-Koh2023-ok)] (see Multimedia Appendix 3): the primary questions RQ1 and RQ4 were combined into the primary RQ in the main text above, and the rest of the questions (RQ2, RQ3, RQ5) were answered here.

We extracted information from various sources through non-systematic secondary literature searches to elaborate on the properties of instruments included in Table 1 in the main text, through the following research questions previously defined in our protocol:

- **Research Question 2 (RQ2)**: What is the format (e.g., number of items, response format) and structure (e.g., sub-scales, dimensionality) of the instruments identified in RQ?
- **Research Question 3 (RQ3)**: What are the core psychometric properties (e.g., reliability, validity, responsiveness, and norms used) of the instruments identified in RQ (assessed in the original publication and other relevant papers)?
- **Research Question 5 (RQ5)**: Which mental health construct(s) are the instruments identified in RQ intended to measure in the identified empirical study (as described in the empirical study), and which mental health construct(s) were the instruments originally developed to measure?

In-depth psychometric evaluation of instruments found in RQ is beyond the scope of this review. However, to ensure that this audit is useful to future researchers, an initial evaluation and literature scoping were performed. We believe the groundwork done in this review can lead to a natural progression for future researchers who are interested in using existing gold-standard instruments to conduct COnsensus-based Standards for the selection of health Measurement INstruments (COSMIN) [[3](#ref-Mokkink2010-xw)] studies based on the results from this review.

### Research Question 2

*RQ2: What is the format (e.g., number of items, response format) and structure (e.g., sub-scales, dimensionality) of the instruments identified in RQ?*

The rest of the research questions were answered using secondary literature searches (non-systematic). Table S2 summarises the structure and format of the 24 instruments according to their original publications. Most instruments are in the public domain, with some restricted to non-commercial use or research purposes only. The number of items on the instruments varies from 4 to 40, with multi-dimensional scales naturally tending to have more items. All instruments adopted rating scales (from 4-point to 7-point scales) except the EQ-5D, which includes an additional visual analog scale [[4](#ref-Brooks1996-ub)]. The measured duration or recall period of these instruments varies, including present moment, past week, past two weeks, past one month, and past year. SWL measures an individual’s life satisfaction without a specific recall period.

The focus of this review on measurement across time is important because the desirable instrument format (e.g., number of response options, number of questions) differs between instruments measuring multiple time points versus once-off [[5](#ref-Hyland2003-lh)]. For example, participant burden concerns mean that longitudinal measures should contain a smaller number of question items (between 1-40 items) with multiple response options (e.g., 5-point Likert scales), compared to cross-sectional measures, which typically contain a larger number of question items (between 20-100 items) with more limited response options [[5](#ref-Hyland2003-lh)]. The results show that the structure and format of all selected instruments align with the desirable properties of a longitudinal measure, that is, the number of items is less than or equal to 40 and there are multiple response options between 4-point and 7-point rating scales [[6](#ref-Guyatt1987-ub)].

**Table S2** *Structure and format of the 24 synthesized instruments in this review that measure state-like mental health constructs and were being administered digitally and repeatedly across time in empirical studies.*

| Instrument | Public Domain | Dimensionality | Number of Items | Response Format | Duration |
| --- | --- | --- | --- | --- | --- |
| PHQ-9 | Yes | Unidimensional | 9 | 4-point scale from 0 (not at all) to 3 (nearly every day) | past 2 weeks |
| GAD-7 | Yes | Unidimensional | 7 | 4-point scale from 0 (not at all) to 3 (nearly every day) | past 2 weeks |
| DASS-21 | Yes | Multidimensional | 21 | 4-point scale from 0 (did not apply to me at all) to 3 (applied to me very much, or most of the time) | past week |
| K10 | Yes | Unidimensional | 10 | 5-point scale from 1 (none of the time) to 5 (all of the time) | past 4 weeks |
| PSS-10 | Yes^a^ | Multidimensional | 10 | 5-point scale from 0 (never) to 4 (very often) | past month |
| HADS | No | Multidimensional | 14 | 4-point scale from 0 to 3 | past week |
| BDI-II | No | Unidimensional | 21 | 4-point scale from 0 to 3 | past 2 weeks |
| SWL | Yes | Unidimensional | 5 | 7-point scale from 1 (strongly disagree) to 7 (strongly agree) | general |
| CES-D | Yes | Multidimensional | 20 | 4-point scale from 0 (“Rarely or none of the time [less than 1 day]”) to 3 (“Most or all of the time [5-7 days]”) | past week |
| WEMWBS | Yes^a^ | Unidimensional | 14 | 5-point scale from 1 (none of the time) to 5 (all of the time) | past 2 weeks |
| PHQ-8 | Yes | Unidimensional | 8 | 4-point scale from 0 (not at all) to 3 (nearly every day) | past 2 weeks |
| PANAS | Yes^a^ | Multidimensional | 20 | 5-point scale from 1 (very slightly or not at all) to 5 (extremely) | past week |
| QIDS-SR | Yes^a^ | Unidimensional | 16 | 4-point scale from 0 to 3 | past 7 days |
| AUDIT | Yes | Multidimensional | 10 | 5-point scale from 0 to 4 | past 12 months |
| EDE-Q | Yes^a^ | Multidimensional | 36 (original version), 28 (newer version) | 7-point scale and some manual inputs | past 28 days |
| IES-R | Yes | Multidimensional | 22 | 5-point scale from 0 (not at all) to 4 (extremely) | past 7 days |
| SF-36 | Yes | Multidimensional | 36 (Not all are mental health related) | 5-point scales (questions related to mental health) | past 4 weeks |
| PDSS-SR | Yes^a^ | Unidimensional? | 7 | 5-point scale (with 0 denoting no symptoms, and higher ratings reflecting greater symptom severity). | past week |
| PSS-4 | Yes^a^ | Unidimensional | 4 | 5-point scale from 0 (never) to 4 (very often) | past month |
| PSS-14 | Yes^a^ | Multidimensional | 14 | 5-point scale from 0 (never) to 4 (very often) | past month |
| WHOQOL-BREF | Free for students only | Multidimensional | 6 (Psychological) + 3 (Social relationship), Total: 26 items | 5-point scale from 1 (not at all) to 5 (extremely) (questions related to mental health including social relationship) | past 2 weeks |
| EQ-5D | Yes^a^ | Multidimensional | 5 + 1 (VAS) - Full instrument, Anxiety/depression - 1 | 3-point scale from 1 (no problem) to 3 (major problems), and 1 Visual Analogue Scale | today |
| MHC-SF | Yes^a^ | Multidimensional | 14 | 6-point scale from “never” to “every day.” | past month |
| STAI | No | Multidimensional | 40 (State: 20, Trait: 20) | 4-point scale from 1 (not at all) to 4 (very much so) | present (state); general (trait) |

*Note*. ^a^ terms of use are free for non-profit/non-commercial/research use only.

### Research Question 3

*RQ3: What are the core psychometric properties (e.g., reliability, validity, responsiveness, and norms used) of the instruments identified in RQ (assessed in the original publication and other relevant papers)?*

Table S3 shows the core psychometric properties of the 24 instruments based on the rating systems and quality criteria defined by Terwee and colleagues [[7](#ref-Terwee2007-bs)], briefly defined below.

- **Content validity** concerns how comprehensively the items in an instrument cover the domain of interest.
- **Construct validity** concerns how the scores on an instrument relate to other instruments that measure the same construct, derived through hypotheses.
- **Criterion validity** concerns how the scores on an instrument relate to a gold standard such as structured clinical interviews based on diagnostic manuals.
- **Internal consistency reliability** concerns the inter-correlated relationship between items within a scale or sub-scale, indicating measuring the same construct.
- **Test-retest reliability** concerns the consistency of an instrument’s scores on repeated measures under the same condition, commonly indicated by the correlation between the first and second (or subsequent) score(s) or Intraclass Correlation Coefficient [[8](#ref-Berchtold2016-ae)].
- **Responsiveness (or sensitivity to change)** concerns the ability of an instrument to detect important changes over time.
- **Floor and ceiling effects** concern the number or percentage of respondents who achieve the lowest (floor) or highest (ceiling) possible score. The effect is defined as > 15% of participants selecting the lowest possible (floor) or highest possible (ceiling) response [[7](#ref-Terwee2007-bs)].
- **Normative data** represents a reference sample from which a scale is calibrated to derive standard scores. The normative data/sample typically consists of a group of individuals representative of the broader population.

The psychometric properties were collected from the original publication describing the instrument, followed by non-systematic secondary searches of any systematic review, meta-analysis or study that evaluated the psychometric properties of the instruments (in all modalities^[[1]](#footnote-1)^). All included studies in Table S3 administered the English version of the instruments to the general adult population, either to the general public (GP) or to primary care patients/outpatients (PC/OP).

**Table S3** *The psychometric properties of the 24 instruments based on the original publication and other studies*

| Instrument | Content Validity | Internal Consistency | Test-retest Reliability | Criterion Validity | Construct Validity | Responsiveness | Floor and ceiling effects | Normative Data |
| --- | --- | --- | --- | --- | --- | --- | --- | --- |
| PHQ-9 | + [[9](#ref-Kroenke2001-sp)] | + [[10](#ref-Patten2009-qk)–[12](#ref-Manea2015-kx)] | ? (PC/OP) [[9](#ref-Kroenke2001-sp)] | + [[9](#ref-Kroenke2001-sp),[11](#ref-Adewuya2006-ct),[13](#ref-Kroenke2010-eu)] | ? (PC/OP) [[9](#ref-Kroenke2001-sp)] | + (PC/OP) [[13](#ref-Kroenke2010-eu)–[16](#ref-Lowe2004-eo)] | + (PC/OP) [[17](#ref-Amtmann2014-rt)]; - (PC/OP) [[18](#ref-Marrie2024-vw)]; ? (GP) [[19](#ref-Cannon2007-yi)] | UK [[20](#ref-Gilbody2007-md)], Australia [[21](#ref-Stafford2007-xc)–[24](#ref-Pirkis2009-mk)], USA [[25](#ref-Thombs2008-xk),[26](#ref-Williams2005-ay)], Canada [[10](#ref-Patten2009-qk)] |
| GAD-7 | + [[27](#ref-Spitzer2006-lk)] | + (PC/OP) [[27](#ref-Spitzer2006-lk)] | + (PC/OP) [[27](#ref-Spitzer2006-lk)] | + [[27](#ref-Spitzer2006-lk),[28](#ref-Plummer2016-td)] | ? (PC/OP) [[27](#ref-Spitzer2006-lk)] | ? (PC/OP) [[13](#ref-Kroenke2010-eu),[29](#ref-Kroenke2009-yx),[30](#ref-Kroenke2007-hg)] | - (PC/OP) [[31](#ref-Richardson2017-qy)]; - (GP) [[32](#ref-Tomitaka2021-nf)] | UK [[33](#ref-Stochl2020-aq)], USA [[27](#ref-Spitzer2006-lk)], Australia [[22](#ref-Stocker2021-bh),[23](#ref-Kiely2015-dn)] |
| DASS-21 | + [[34](#ref-Lovibond1995-hm)] | + [[35](#ref-Antony1998-ef)–[37](#ref-Osman2012-al)] | ? (GP) [[38](#ref-Zlomke2009-sa)] | ? [[34](#ref-Lovibond1995-hm),[35](#ref-Antony1998-ef),[39](#ref-Norton2007-rp)] | + (PC/OP) [[40](#ref-Gloster2008-th)]; ? (GP) [[36](#ref-Henry2005-yf)] | ? (PC/OP) [[41](#ref-Yohannes2019-ij)] | - (GP) [[42](#ref-Sinclair2012-so)]; + (PC/OP) [[43](#ref-Park2020-lq)] | Australia [[44](#ref-Crawford2011-tl)], UK [[36](#ref-Henry2005-yf)], USA [[42](#ref-Sinclair2012-so)] |
| K10 | + [[46](#ref-Kessler2002-qx)] | + [[46](#ref-Kessler2002-qx)–[48](#ref-Kessler2003-fp)] | + [[49](#ref-Furukawa2003-lg),[50](#ref-Merson2021-am),[50](#ref-Merson2021-am)] | + [[47](#ref-Hides2007-sk),[49](#ref-Furukawa2003-lg)] | ? (GP) [[48](#ref-Kessler2003-fp)] | + (PC/OP) [[51](#ref-Perini2006-nd)] | - (GP) [[52](#ref-Andrews2001-kk),[53](#ref-Rahman2021-gq)] | US [[46](#ref-Kessler2002-qx)], Australia [[49](#ref-Furukawa2003-lg),[54](#ref-Slade2011-ib)–[56](#ref-Stallman2010-cj)] |
| PSS-10 | + [[57](#ref-Cohen1983-gk),[58](#ref-Cohen1988-nn)] | + (GP) [[59](#ref-Ezzati2014-ov),[60](#ref-Lee2012-wp)] | 0 | ? (GP) [[60](#ref-Lee2012-wp),[61](#ref-Mitchell2008-mf)] | + (GP) [[58](#ref-Cohen1988-nn)]; ? (GP) [[61](#ref-Mitchell2008-mf)] | 0 | - (PC/OP) [[62](#ref-Wiriyakijja2020-wr)]; + (GP) [[63](#ref-Anwer2020-rf)] | US [[58](#ref-Cohen1988-nn),[59](#ref-Ezzati2014-ov)], Australian pharmacists [[64](#ref-Chapman2020-uc)] |
| HADS | + [[65](#ref-Zigmond1983-dj)] | + [[66](#ref-Bjelland2002-ef),[67](#ref-Crawford2001-rc)] | ? (PC/OP) [[68](#ref-McPherson2011-jv),[69](#ref-Karimova2003-ft)] | + [[66](#ref-Bjelland2002-ef),[70](#ref-Herrmann1997-zz)] | ? (PC/OP) [[71](#ref-Cameron2008-jb)] | ? (PC/OP) [[71](#ref-Cameron2008-jb)] | ? (GP) [[67](#ref-Crawford2001-rc)] | UK [[65](#ref-Zigmond1983-dj),[67](#ref-Crawford2001-rc)] |
| BDI-II | + [[72](#ref-Beck1996-au)] | + [[73](#ref-Dozois1998-qh)] | ? (GP) [[74](#ref-Wang2013-ql)] | + [[74](#ref-Wang2013-ql)] | + [[74](#ref-Wang2013-ql)] | 0 | 0 | US [[72](#ref-Beck1996-au),[75](#ref-Carmody2005-ze),[76](#ref-Whisman2015-eg)], Australia [[44](#ref-Crawford2011-tl)] |
| SWLS | + [[77](#ref-Diener1985-ka)] | + (GP) [[77](#ref-Diener1985-ka),[78](#ref-Van_Agteren2021-iu)] | ? (GP) [[77](#ref-Diener1985-ka)] | ? (GP) [[77](#ref-Diener1985-ka)] | ? (GP) [[77](#ref-Diener1985-ka),[79](#ref-van-Beuningen2012-ym)–[81](#ref-Pavot1991-lk)] | ? (GP) [[82](#ref-Pavot2008-ju)] | + [[83](#ref-Hagell2013-vr),[84](#ref-Rosengren2015-ru)] | USA [[77](#ref-Diener1985-ka),[85](#ref-Cheung2014-ni)] See Table 1 of [[80](#ref-Pavot1993-te)]. |
| CES-D | + [[86](#ref-Radloff1977-vh)] | + (GP) [[86](#ref-Radloff1977-vh),[87](#ref-Mongrain2018-uc)] | ? (GP) [[86](#ref-Radloff1977-vh)] | + [[88](#ref-Vilagut2016-yw)] | ? (GP) [[86](#ref-Radloff1977-vh)]; + (PC/OP) [[89](#ref-Hann1999-hr)] | 0 | + (PC/OP) [[17](#ref-Amtmann2014-rt)]; - (GP) [[90](#ref-Wilson2004-at)] | US [[86](#ref-Radloff1977-vh)]; Australia [[44](#ref-Crawford2011-tl),[91](#ref-Christensen2011-xy)] |
| WEMWBS | + [[92](#ref-Tennant2007-to)] | + (GP) [[92](#ref-Tennant2007-to),[93](#ref-Lloyd2012-zk)] | + (GP) [[92](#ref-Tennant2007-to)] | ? (GP) [[92](#ref-Tennant2007-to),[93](#ref-Lloyd2012-zk)] | + (GP) [[92](#ref-Tennant2007-to)]; ? (GP) [[93](#ref-Lloyd2012-zk)] | + [[94](#ref-Maheswaran2012-tm)] | + [[92](#ref-Tennant2007-to),[94](#ref-Maheswaran2012-tm)] | UK [[92](#ref-Tennant2007-to)]; Mixed [[94](#ref-Maheswaran2012-tm)] |
| PHQ-8 | + [[95](#ref-Kroenke2009-zx),[96](#ref-Dancyger2022-ja)] | + [[97](#ref-Thakur2018-hr),[98](#ref-Jin2020-zy)] | + (PC/OP) [[98](#ref-Jin2020-zy)] | + [[95](#ref-Kroenke2009-zx),[99](#ref-Wu2020-oy)] | ? (GP) [[95](#ref-Kroenke2009-zx)] | 0 | - (GP) [[32](#ref-Tomitaka2021-nf)] | US [[95](#ref-Kroenke2009-zx),[98](#ref-Jin2020-zy)]; UK [[96](#ref-Dancyger2022-ja)]; Mixed [[99](#ref-Wu2020-oy)] |
| PANAS | + [[100](#ref-Watson1988-gn)] | + (GP) [[100](#ref-Watson1988-gn)] | ? (GP) [[100](#ref-Watson1988-gn)] | 0 | + (GP) [[101](#ref-Crawford2004-jc)] | 0 | 0 | US [[100](#ref-Watson1988-gn)]; UK [[101](#ref-Crawford2004-jc)] |
| QIDS-SR | + [[102](#ref-Rush1996-qz),[103](#ref-Rush2003-hu)] | + [[103](#ref-Rush2003-hu)–[106](#ref-Trivedi2004-wu)] | 0 [[105](#ref-Reilly2015-bd)] | ? (PC/OP) [[103](#ref-Rush2003-hu)] | ? [[103](#ref-Rush2003-hu),[105](#ref-Reilly2015-bd)] | ? [[103](#ref-Rush2003-hu),[105](#ref-Reilly2015-bd),[107](#ref-Lamoureux2010-cr)] | 0 | US [[103](#ref-Rush2003-hu)]; UK [[108](#ref-Cameron2013-co)] |
| AUDIT | + [[109](#ref-Saunders1993-xs)] | + [[109](#ref-Saunders1993-xs),[110](#ref-Babor2001-rc)] | ? (GP) [[110](#ref-Babor2001-rc)]; ? (PC/OP) [[111](#ref-Reinert2002-xs),[112](#ref-Maisto2000-yf)] | + [[113](#ref-Allen1997-oz),[114](#ref-Clements1998-wk)] | ? [[109](#ref-Saunders1993-xs),[113](#ref-Allen1997-oz)] | 0 | ? (GP) [[115](#ref-Peng2012-oj)] | Mixed [[113](#ref-Allen1997-oz)] |
| EDE-Q | + [[116](#ref-Fairburn1994-zj)] | + (GP) [[117](#ref-Mond2006-tf),[118](#ref-Luce1999-uw)] | ? (GP) [[117](#ref-Mond2006-tf),[118](#ref-Luce1999-uw)]; ? (PC/OP) [[119](#ref-Berg2012-sj)] | + (GP) [[120](#ref-Mond2004-wk)] | ? [[119](#ref-Berg2012-sj),[121](#ref-Rosen1990-as)] | 0 | 0 | Australia [[117](#ref-Mond2006-tf)]; US [[122](#ref-Jennings2017-et)] |
| IES-R | + [[123](#ref-Weiss1997-gx),[124](#ref-Weiss2004-xp)] | + [[124](#ref-Weiss2004-xp),[125](#ref-Creamer2003-ar)] | ? (GP) [[123](#ref-Weiss1997-gx)–[126](#ref-Adkins2008-sm)] | ? [[125](#ref-Creamer2003-ar),[127](#ref-Beck2008-ux)] | + (GP) [[125](#ref-Creamer2003-ar),[126](#ref-Adkins2008-sm)] | 0 | 0 | US [[123](#ref-Weiss1997-gx)–[125](#ref-Creamer2003-ar)] |
| SF-36 | + [[128](#ref-Ware1994-em),[129](#ref-Ware1992-bd)] | + [[130](#ref-Butterworth2004-xd),[131](#ref-Brazier1992-ug)] | ? (PC/OP) [[131](#ref-Brazier1992-ug)] | ? [[130](#ref-Butterworth2004-xd),[132](#ref-Jenkinson1994-zn),[133](#ref-Stansfeld1997-nb)] | + [[130](#ref-Butterworth2004-xd),[131](#ref-Brazier1992-ug)] | + (PC/OP) [[134](#ref-Garratt1994-ws),[135](#ref-Katz1992-aj)] | - (GP) [[136](#ref-Thumboo2001-ml)] | Australia [[130](#ref-Butterworth2004-xd)]; US [[128](#ref-Ware1994-em)]; UK [[131](#ref-Brazier1992-ug),[137](#ref-Jenkinson1993-yc)] |
| PDSS-SR | + [[138](#ref-Houck2002-qk)] | + (PC/OP) [[138](#ref-Houck2002-qk),[139](#ref-Hadjistavropoulos2019-lp)]; - (PC/OP) [[140](#ref-Shear1997-yq)] | + (PC/OP) [[138](#ref-Houck2002-qk)] | + (PC/OP) [[140](#ref-Shear1997-yq)] | ? (PC/OP) [[140](#ref-Shear1997-yq)] | ? (PC/OP) [[138](#ref-Houck2002-qk),[140](#ref-Shear1997-yq)] | 0 | US [[138](#ref-Houck2002-qk),[140](#ref-Shear1997-yq)]; Australia [[141](#ref-Newby2014-or)] |
| PSS-4 | + [[58](#ref-Cohen1988-nn)] | - (GP) [[58](#ref-Cohen1988-nn),[59](#ref-Ezzati2014-ov)]; + (GP) [[142](#ref-Warttig2013-fg)] | 0 | ? (GP) [[61](#ref-Mitchell2008-mf)] | ? (GP) [[61](#ref-Mitchell2008-mf)] | 0 | + (GP) [[143](#ref-Wu2013-vf)] | UK [[142](#ref-Warttig2013-fg)]; US [[58](#ref-Cohen1988-nn)]; US 70+ [[59](#ref-Ezzati2014-ov)] |
| PSS-14 | + [[57](#ref-Cohen1983-gk),[58](#ref-Cohen1988-nn)] | + (GP) [[59](#ref-Ezzati2014-ov),[61](#ref-Mitchell2008-mf),[144](#ref-Goldberg2020-hm)] | ? (GP) [[57](#ref-Cohen1983-gk)] | ? (GP) [[61](#ref-Mitchell2008-mf),[145](#ref-Ribeiro-Santiago2020-sq)] | ? (GP) [[[61](#ref-Mitchell2008-mf)]; ] | 0 | + (GP) [[143](#ref-Wu2013-vf)] | US [[57](#ref-Cohen1983-gk),[59](#ref-Ezzati2014-ov)]; Australia (Aboriginal) [[146](#ref-Ribeiro_Santiago2020-yc)] |
| WHOQOL-BREF | + [[147](#ref-Skevington2004-mv)] | - (GP, PC/OP) [[147](#ref-Skevington2004-mv)]; + (GP) [[148](#ref-Skevington2012-kx),[149](#ref-Suarez2018-dw)] | ? (GP) [[147](#ref-Skevington2004-mv),[148](#ref-Skevington2012-kx)] | ? [[148](#ref-Skevington2012-kx),[149](#ref-Suarez2018-dw)] | ? [[148](#ref-Skevington2012-kx),[149](#ref-Suarez2018-dw)] | ? [[148](#ref-Skevington2012-kx)] | + [[147](#ref-Skevington2004-mv)] | Mixed [[147](#ref-Skevington2004-mv)]; Singapore [[149](#ref-Suarez2018-dw)]; UK [[148](#ref-Skevington2012-kx)] |
| EQ-5D | + [[150](#ref-EuroQol_Group1990-ns)] | - (GP) [[151](#ref-Khanna2013-vw),[152](#ref-Zakershahrak2022-ab)] | + (GP) [[4](#ref-Brooks1996-ub),[153](#ref-Dolan1994-ni)] | ? [[4](#ref-Brooks1996-ub),[152](#ref-Zakershahrak2022-ab)] | + (GP) [[151](#ref-Khanna2013-vw),[154](#ref-Brazier1993-ad)] | 0 | - (GP) [[152](#ref-Zakershahrak2022-ab),[154](#ref-Brazier1993-ad)] | Australia [[152](#ref-Zakershahrak2022-ab)]; UK [[154](#ref-Brazier1993-ad)]; Mixed [[155](#ref-Janssen2013-cx)] |
| MHC-SF | + [[156](#ref-Keyes2005-bh)] | + (GP) [[157](#ref-Levin2017-fm),[158](#ref-Pir2021-fq)] | 0 | ? (GP) [[159](#ref-Orpana2017-rb)] | ? (GP) [[159](#ref-Orpana2017-rb)] | 0 | 0 | US [[156](#ref-Keyes2005-bh)]; New Zealand [[158](#ref-Pir2021-fq)]; Canada [[159](#ref-Orpana2017-rb)]; Mixed [[160](#ref-Iasiello2022-nu),[161](#ref-Weiss2016-ey)]; Australia [[162](#ref-Hides2016-gq)] |
| STAI | + [[163](#ref-Spielberger1983-iv),[164](#Xfaa0c7bf27e1c1d0ab6e98c75773f4c702636c5)] | + [[163](#ref-Spielberger1983-iv)–[165](#ref-Barnes2002-uq)] | ? [[164](#Xfaa0c7bf27e1c1d0ab6e98c75773f4c702636c5)–[166](#ref-Shahid2012-ub)] | ? [[164](#Xfaa0c7bf27e1c1d0ab6e98c75773f4c702636c5)] | ? [[164](#Xfaa0c7bf27e1c1d0ab6e98c75773f4c702636c5),[167](#ref-Balsamo2013-qr)] | ? [[168](#ref-Julian2011-yv)] | + [[169](#ref-Santangelo2016-zl)] | US [[163](#ref-Spielberger1983-iv)]; Australia [[44](#ref-Crawford2011-tl)] |

*Note*. +: positive rating; ?: indeterminate rating; -: negative rating; 0: no information available; GP: General population; PC/OP: Primary Care/Outpatients

Our evaluation of the psychometric properties (as the quality assessment component) of 24 instruments was only provisional, and conducted through non-systematic reviews (for a similar strategy, see [[172](#ref-Breedvelt2020-hp)]). Given the aims and logic of this review, the provisional evaluation of psychometric properties presented above should be interpreted with caution. This review follows the quality criteria suggested by Terwee and colleagues [[7](#ref-Terwee2007-bs)]. Readers are encouraged to cross-check these evaluations with other psychometric evaluations that use other evaluation frameworks. Future systematic reviews focusing on the evaluation of psychometric properties of outcome measurements are encouraged to use standards such as COSMIN [[3](#ref-Mokkink2010-xw)] and the emerging PRISMA-COSMIN reporting guidelines [[173](#ref-Elsman2024-kt)].

The observation that some included studies administered instruments measuring mental health constructs multiple times a day, as seen in EMA studies, raises further questions about potential measurement bias and the psychometric properties of the instruments when they are used in repeated measurements between short intervals. For example, the *practice effect* [[174](#ref-Coulacoglou2017-pz),[175](#ref-Schmitter-Edgecombe2020-zt)] is a known bias in psychological testing where individuals’ performance improved on the same test when it was repeatedly administered. Although the practice effect is more apparent and detrimental in cognitive tests [[176](#ref-Collie2003-pd)–[178](#ref-Chen2023-gf)], self-report items capturing mental health indicators may suffer from similar bias [[179](#ref-Achtyes2015-dd)] when the same items or tests were repeatedly administered in short intervals, which consequently, could affect the inference made [[180](#ref-McAleavey2024-cb)].

While ubiquitous digital technologies have enabled us to collect mental health data more feasibly and regularly than ever before, there is a potential gap in the literature about the implications of measuring mental health under this specific context with the digital version of existing commonly used instruments, which were originally developed and evaluated for paper-and-pen format and having longer intervals between measurements.

### Research Question 5

*RQ5: Which mental health construct(s) are the instruments identified in RQ intended to measure in the identified empirical study (as described in the empirical study), and which mental health construct(s) were the instruments originally developed to measure?*

Table S4 lists the constructs the identified instruments were originally developed to measure and any deviations from these constructs found in later studies using the instrument in the 223 identified empirical studies. We did not identify any substantial mismatches, but a lack of conceptual precision was observed in the use of 15 instruments. For example, K-10, a measure of psychological distress, was used in some studies to measure negative emotions or (absence of) psychological well-being. Similarly, PHQ-9 and GAD-7 were originally developed to measure symptoms of clinical depression and anxiety respectively, and were used as indicators of mood or negative affect. Finally, SWL was developed to measure satisfaction with life but was in some cases used as an indicator of general well-being.

**Table S4** *The original mental health construct(s) measured and observed variations in later empirical studies.*

| **Instrument** | **Constructs (Original)** | **Constructs (Variations from Empirical Studies)** |
| --- | --- | --- |
| PHQ-9 | Depressive symptoms based on DSM-IV | (1) Part of the indicators of negative affect or mood. (2) Question 9 of this instrument was used to measure severity of suicidal ideation or suicide risk. |
| GAD-7 | Generalised Anxiety Disorder symptoms based on DSM-IV | (1) Part of the indicators of negative affect or mood. (2) Postpartum anxiety |
| DASS-21 | Symptoms of depression, anxiety and stress | (1) Psychological distress (2) Negative emotional states / Mood |
| K10 | Psychological Distress | Negative emotions experienced / Psychological well-being |
| PSS-10 | Psychological Stress (Perceived Helplessness and Perceived Self-Efficacy) | Stress Level (the degree to which a situation in someone’s life is considered stressful) |
| BDI-II | Severity of depressive symptomatology | Severity of suicidal ideation / suicide risk (Item 9) |
| SWL | Global life satisfaction | General well-being |
| CES-D | Depressive symptomatology | Mood |
| IES-R | PTSD symptoms (avoidance, intrusions, hyperarousal) | Subjective distress caused by a traumatic event |
| SF-36 | Physical functioning, role physical, bodily pain, general health, vitality, social functioning, role emotional, and mental health | Emotional Well-being (mental health component) |
| PSS-14 | Psychological Stress | Perception of stressful events / appraisals of stress |
| PSS-4 | Psychological Stress | (1) Perceived Stress (2) An individual’s confidence in handling problems |
| WHOQOL-BREF | Quality of Life | Health-Related Quality of Life |
| EQ-5D | Quality of Life | Health-Related Quality of Life |
| MHC-SF | Mental well-being (emotional well-being, psychological well-being and social well-being) | (1) Positive mental health (2) Flourishing |

# References

1. Koh ZH, Skues J, Murray G. Digital self-report instruments for repeated measurement of mental health in the general adult population: A protocol for a systematic review. BMJ open 2023 Jan;13(1):e065162. PMID:[36693693](https://www.ncbi.nlm.nih.gov/pubmed/36693693)

2. Beidas RS, Stewart RE, Walsh L, Lucas S, Downey MM, Jackson K, Fernandez T, Mandell DS. Free, brief, and validated: Standardized instruments for low-resource mental health settings. Cognitive and behavioral practice 2015 Feb;22(1):5–19. PMID:[25642130](https://www.ncbi.nlm.nih.gov/pubmed/25642130)

3. Mokkink LB, Terwee CB, Patrick DL, Alonso J, Stratford PW, Knol DL, Bouter LM, De Vet HCW. The COSMIN checklist for assessing the methodological quality of studies on measurement properties of health status measurement instruments: An international Delphi study. Quality of life research 2010;19(4):539–549. doi: [10.1007/s11136-010-9606-8](https://doi.org/10.1007/s11136-010-9606-8)

4. Brooks R. EuroQol: The current state of play. Health policy 1996 Jul;37(1):53–72. PMID:[10158943](https://www.ncbi.nlm.nih.gov/pubmed/10158943)

5. Hyland ME. A brief guide to the selection of quality of life instrument. Health and quality of life outcomes 2003 Jul;1:24. PMID:[12848895](https://www.ncbi.nlm.nih.gov/pubmed/12848895)

6. Guyatt G, Walter S, Norman G. Measuring change over time: Assessing the usefulness of evaluative instruments. Journal of chronic diseases Elsevier; 1987;40(2):171–178. PMID:[3818871](https://www.ncbi.nlm.nih.gov/pubmed/3818871)

7. Terwee CB, Bot SDM, Boer MR de, Windt DAWM van der, Knol DL, Dekker J, Bouter LM, Vet HCW de. Quality criteria were proposed for measurement properties of health status questionnaires. Journal of clinical epidemiology Elsevier; 2007 Jan;60(1):34–42. PMID:[17161752](https://www.ncbi.nlm.nih.gov/pubmed/17161752)

8. Berchtold A. Test–retest: Agreement or reliability? Methodological innovations SAGE Publications; 2016 Jan;9:205979911667287. doi: [10.1177/2059799116672875](https://doi.org/10.1177/2059799116672875)

9. Kroenke K, Spitzer RL, Williams JB. The PHQ-9: Validity of a brief depression severity measure. Journal of general internal medicine 2001 Sep;16(9):606–613. PMID:[11556941](https://www.ncbi.nlm.nih.gov/pubmed/11556941)

10. Patten SB, Schopflocher D. Longitudinal epidemiology of major depression as assessed by the brief Patient Health Questionnaire (PHQ-9). Comprehensive psychiatry 2009 Jan;50(1):26–33. PMID:[19059510](https://www.ncbi.nlm.nih.gov/pubmed/19059510)

11. Adewuya AO, Ola BA, Afolabi OO. Validity of the Patient Health Questionnaire (PHQ-9) as a screening tool for depression amongst Nigerian university students. Journal of affective disorders 2006 Nov;96(1-2):89–93. PMID:[16857265](https://www.ncbi.nlm.nih.gov/pubmed/16857265)

12. Manea L, Gilbody S, McMillan D. A diagnostic meta-analysis of the Patient Health Questionnaire-9 (PHQ-9) algorithm scoring method as a screen for depression. General hospital psychiatry Elsevier BV; 2015 Jan;37(1):67–75. PMID:[25439733](https://www.ncbi.nlm.nih.gov/pubmed/25439733)

13. Kroenke K, Spitzer RL, Williams JBW, Löwe B. The patient health questionnaire somatic, anxiety, and depressive symptom scales: A systematic review. General hospital psychiatry 2010 Jul;32(4):345–359. PMID:[20633738](https://www.ncbi.nlm.nih.gov/pubmed/20633738)

14. Williams LS, Kroenke K, Bakas T, Plue LD, Brizendine E, Tu W, Hendrie H. Care management of poststroke depression: A randomized, controlled trial. Stroke 2007 Mar;38(3):998–1003. PMID:[17303771](https://www.ncbi.nlm.nih.gov/pubmed/17303771)

15. Löwe B, Unützer J, Callahan CM, Perkins AJ, Kroenke K. Monitoring depression treatment outcomes with the Patient Health Questionnaire-9. Medical care 2004 Dec;42(12):1194–1201. PMID:[15550799](https://www.ncbi.nlm.nih.gov/pubmed/15550799)

16. Löwe B, Kroenke K, Herzog W, Gräfe K. Measuring depression outcome with a brief self-report instrument: Sensitivity to change of the Patient Health Questionnaire (PHQ-9). Journal of affective disorders Elsevier BV; 2004 Jul;81(1):61–66. PMID:[15183601](https://www.ncbi.nlm.nih.gov/pubmed/15183601)

17. Amtmann D, Kim J, Chung H, Bamer AM, Askew RL, Wu S, Cook KF, Johnson KL. Comparing CESD-10, PHQ-9, and PROMIS depression instruments in individuals with multiple sclerosis. Rehabilitation psychology American Psychological Association (APA); 2014 May;59(2):220–229. PMID:[24661030](https://www.ncbi.nlm.nih.gov/pubmed/24661030)

18. Marrie RA, Fisk JD, Dolovich C, Lix LM, Graff LA, Patten SB, Bernstein CN. Psychometric performance of fatigue scales in inflammatory bowel disease. Inflammatory bowel diseases Oxford University Press; 2024 Jan;30(1):53–63. PMID:[36917218](https://www.ncbi.nlm.nih.gov/pubmed/36917218)

19. Cannon DS, Tiffany ST, Coon H, Scholand MB, McMahon WM, Leppert MF. The PHQ-9 as a brief assessment of lifetime major depression. Psychological assessment American Psychological Association (APA); 2007 Jun;19(2):247–251. PMID:[17563207](https://www.ncbi.nlm.nih.gov/pubmed/17563207)

20. Gilbody S, Richards D, Barkham M. Diagnosing depression in primary care using self-completed instruments: UK validation of PHQ-9 and CORE-OM. The British journal of general practice 2007 Aug;57(541):650–652. PMID:[17688760](https://www.ncbi.nlm.nih.gov/pubmed/17688760)

21. Stafford L, Berk M, Jackson HJ. Validity of the Hospital Anxiety and Depression Scale and Patient Health Questionnaire-9 to screen for depression in patients with coronary artery disease. General hospital psychiatry 2007 Sep;29(5):417–424. PMID:[17888808](https://www.ncbi.nlm.nih.gov/pubmed/17888808)

22. Stocker R, Tran T, Hammarberg K, Nguyen H, Rowe H, Fisher J. Patient Health Questionnaire 9 (PHQ-9) and General Anxiety Disorder 7 (GAD-7) data contributed by 13,829 respondents to a national survey about COVID-19 restrictions in Australia. Psychiatry research 2021 Apr;298:113792. PMID:[33592399](https://www.ncbi.nlm.nih.gov/pubmed/33592399)

23. Kiely KM, Butterworth P. Validation of four measures of mental health against depression and generalized anxiety in a community based sample. Psychiatry research 2015 Feb;225(3):291–298. PMID:[25578983](https://www.ncbi.nlm.nih.gov/pubmed/25578983)

24. Pirkis J, Pfaff J, Williamson M, Tyson O, Stocks N, Goldney R, Draper B, Snowdon J, Lautenschlager N, Almeida OP. The community prevalence of depression in older Australians. Journal of affective disorders 2009 May;115(1-2):54–61. PMID:[18817976](https://www.ncbi.nlm.nih.gov/pubmed/18817976)

25. Thombs BD, Ziegelstein RC, Whooley MA. Optimizing detection of major depression among patients with coronary artery disease using the Patient Health Questionnaire: Data from the heart and soul study. Journal of general internal medicine 2008 Dec;23(12):2014–2017. PMID:[18815842](https://www.ncbi.nlm.nih.gov/pubmed/18815842)

26. Williams LS, Brizendine EJ, Plue L, Bakas T, Tu W, Hendrie H, Kroenke K. Performance of the PHQ-9 as a screening tool for depression after stroke. Stroke 2005 Mar;36(3):635–638. PMID:[15677576](https://www.ncbi.nlm.nih.gov/pubmed/15677576)

27. Spitzer RL, Kroenke K, Williams JBW, Löwe B. A brief measure for assessing generalized anxiety disorder: The GAD-7. Archives of internal medicine American Medical Association; 2006 May;166(10):1092–1097. doi: [10.1001/archinte.166.10.1092](https://doi.org/10.1001/archinte.166.10.1092)

28. Plummer F, Manea L, Trepel D, McMillan D. Screening for anxiety disorders with the GAD-7 and GAD-2: A systematic review and diagnostic metaanalysis. General hospital psychiatry 2016 Mar;39:24–31. PMID:[26719105](https://www.ncbi.nlm.nih.gov/pubmed/26719105)

29. Kroenke K, Bair MJ, Damush TM, Wu J, Hoke S, Sutherland J, Tu W. Optimized antidepressant therapy and pain self-management in primary care patients with depression and musculoskeletal pain: A randomized controlled trial. JAMA American Medical Association (AMA); 2009 May;301(20):2099–2110. PMID:[19470987](https://www.ncbi.nlm.nih.gov/pubmed/19470987)

30. Kroenke K, Spitzer RL, Williams JBW, Monahan PO, Löwe B. Anxiety disorders in primary care: Prevalence, impairment, comorbidity, and detection. Annals of internal medicine acpjournals.org; 2007 Mar;146(5):317–325. PMID:[17339617](https://www.ncbi.nlm.nih.gov/pubmed/17339617)

31. Richardson T, Wrightman M, Yeebo M, Lisicka A. Reliability and score ranges of the PHQ-9 and GAD-7 in a primary and secondary care mental health service. Journal of Psychosocial Rehabilitation and Mental Health 2017 Dec;4(2):237–240. doi: [10.1007/s40737-017-0090-0](https://doi.org/10.1007/s40737-017-0090-0)

32. Tomitaka S, Furukawa TA. The GAD-7 and the PHQ-8 exhibit the same mathematical pattern of item responses in the general population: Analysis of data from the National Health Interview Survey. BMC psychology Springer Science; Business Media LLC; 2021 Sep;9(1):149. PMID:[34556185](https://www.ncbi.nlm.nih.gov/pubmed/34556185)

33. Stochl J, Fried EI, Fritz J, Croudace TJ, Russo DA, Knight C, Jones PB, Perez J. On dimensionality, measurement invariance, and suitability of sum scores for the PHQ-9 and the GAD-7. Assessment 2020 Dec;29(3):1073191120976863. PMID:[33269612](https://www.ncbi.nlm.nih.gov/pubmed/33269612)

34. Lovibond PF, Lovibond SH. The structure of negative emotional states: Comparison of the Depression Anxiety Stress Scales (DASS) with the Beck Depression and Anxiety Inventories. Behaviour research and therapy 1995 Mar;33(3):335–343. PMID:[7726811](https://www.ncbi.nlm.nih.gov/pubmed/7726811)

35. Antony MM, Bieling PJ, Cox BJ, Enns MW, Swinson RP. Psychometric properties of the 42-item and 21-item versions of the Depression Anxiety Stress Scales in clinical groups and a community sample. Psychological assessment 1998;10(2):176–181. doi: [10.1037/1040-3590.10.2.176](https://doi.org/10.1037/1040-3590.10.2.176)

36. Henry JD, Crawford JR. The short-form version of the Depression Anxiety Stress Scales (DASS-21): Construct validity and normative data in a large non-clinical sample. British journal of clinical psychology Wiley; 2005 Jun;44(2):227–239. PMID:[16004657](https://www.ncbi.nlm.nih.gov/pubmed/16004657)

37. Osman A, Wong JL, Bagge CL, Freedenthal S, Gutierrez PM, Lozano G. The Depression Anxiety Stress Scales-21 (DASS-21): Further examination of dimensions, scale reliability, and correlates. Journal of clinical psychology 2012 Dec;68(12):1322–1338. PMID:[22930477](https://www.ncbi.nlm.nih.gov/pubmed/22930477)

38. Zlomke KR. Psychometric properties of internet administered versions of Penn State Worry Questionnaire (PSWQ) and Depression, Anxiety, and Stress Scale (DASS). Computers in human behavior 2009 Jul;25(4):841–843. doi: [10.1016/j.chb.2008.06.003](https://doi.org/10.1016/j.chb.2008.06.003)

39. Norton PJ. Depression Anxiety and Stress Scales (DASS-21): Psychometric analysis across four racial groups. Anxiety, stress, and coping 2007 Sep;20(3):253–265. PMID:[17999228](https://www.ncbi.nlm.nih.gov/pubmed/17999228)

40. Gloster AT, Rhoades HM, Novy D, Klotsche J, Senior A, Kunik M, Wilson N, Stanley MA. Psychometric properties of the Depression Anxiety and Stress Scale-21 in older primary care patients. Journal of affective disorders 2008 Oct;110(3):248–259. PMID:[18304648](https://www.ncbi.nlm.nih.gov/pubmed/18304648)

41. Yohannes AM, Dryden S, Hanania NA. Validity and responsiveness of the Depression Anxiety Stress Scales-21 (DASS-21) in COPD. Chest Elsevier BV; 2019 Jun;155(6):1166–1177. PMID:[30594559](https://www.ncbi.nlm.nih.gov/pubmed/30594559)

42. Sinclair SJ, Siefert CJ, Slavin-Mulford JM, Stein MB, Renna M, Blais MA. Psychometric evaluation and normative data for the depression, anxiety, and stress scales-21 (DASS-21) in a nonclinical sample of U.S. adults. Evaluation & the health professions 2012 Sep;35(3):259–279. PMID:[22008979](https://www.ncbi.nlm.nih.gov/pubmed/22008979)

43. Park SH, Song YJC, Demetriou EA, Pepper KL, Thomas EE, Hickie IB, Guastella AJ. Validation of the 21-item Depression, Anxiety, and Stress Scales (DASS-21) in individuals with autism spectrum disorder. Psychiatry Research Elsevier; 2020 Sep;291:113300. doi: [10.1016/j.psychres.2020.113300](https://doi.org/10.1016/j.psychres.2020.113300)

44. Crawford J, Cayley C, Lovibond PF, Wilson PH, Hartley C. Percentile norms and accompanying interval estimates from an Australian general adult population sample for self‐report mood scales (BAI, BDI, CRSD, CES‐D, DASS, DASS‐21, STAI‐X, STAI‐Y, SRDS, and SRAS). Australian psychologist Routledge; 2011 Mar;46(1):3–14. doi: [10.1111/j.1742-9544.2010.00003.x](https://doi.org/10.1111/j.1742-9544.2010.00003.x)

45. Crawford JR, Garthwaite PH, Lawrie CJ, Henry JD, MacDonald MA, Sutherland J, Sinha P. A convenient method of obtaining percentile norms and accompanying interval estimates for self-report mood scales (DASS, DASS-21, HADS, PANAS, and sAD). British journal of clinical psychology 2009 Jun;48(2):163–180. PMID:[19054433](https://www.ncbi.nlm.nih.gov/pubmed/19054433)

46. Kessler RC, Andrews G, Colpe LJ, Hiripi E, Mroczek DK, Normand SLT, Walters EE, Zaslavsky AM. Short screening scales to monitor population prevalences and trends in non-specific psychological distress. Psychological medicine Cambridge University Press (CUP); 2002 Aug;32(6):959–976. PMID:[12214795](https://www.ncbi.nlm.nih.gov/pubmed/12214795)

47. Hides L, Lubman DI, Devlin H, Cotton S, Aitken C, Gibbie T, Hellard M. Reliability and validity of the Kessler 10 and Patient Health Questionnaire among injecting drug users. The Australian and New Zealand journal of psychiatry 2007 Feb;41(2):166–168. PMID:[17464695](https://www.ncbi.nlm.nih.gov/pubmed/17464695)

48. Kessler RC, Barker PR, Colpe LJ, Epstein JF, Gfroerer JC, Hiripi E, Howes MJ, Normand S-LT, Manderscheid RW, Walters EE, Zaslavsky AM. Screening for serious mental illness in the general population. Archives of general psychiatry 2003 Feb;60(2):184–189. PMID:[12578436](https://www.ncbi.nlm.nih.gov/pubmed/12578436)

49. Furukawa TA, Kessler RC, Slade T, Andrews G. The performance of the K6 and K10 screening scales for psychological distress in the Australian National Survey of Mental Health and Well-Being. Psychological medicine 2003 Feb;33(2):357–362. PMID:[12622315](https://www.ncbi.nlm.nih.gov/pubmed/12622315)

50. Merson F, Newby J, Shires A, Millard M, Mahoney A. The temporal stability of the Kessler Psychological Distress Scale. Australian psychologist Routledge; 2021 Jan;56(1):38–45. doi: [10.1080/00050067.2021.1893603](https://doi.org/10.1080/00050067.2021.1893603)

51. Perini SJ, Slade T, Andrews G. Generic effectiveness measures: Sensitivity to symptom change in anxiety disorders. Journal of affective disorders 2006 Feb;90(2-3):123–130. PMID:[16337690](https://www.ncbi.nlm.nih.gov/pubmed/16337690)

52. Andrews G, Slade T. Interpreting scores on the Kessler Psychological Distress Scale (K10). Australian and New Zealand journal of public health 2001 Dec;25(6):494–497. PMID:[11824981](https://www.ncbi.nlm.nih.gov/pubmed/11824981)

53. Rahman MA, Salehin M, Islam SMS, Alif SM, Sultana F, Sharif A, Hoque N, Nazim NB, Cross WM. Reliability of the tools used to examine psychological distress, fear of COVID-19 and coping amongst migrants and non-migrants in Australia. International journal of mental health nursing Wiley; 2021 Jun;30(3):747–758. PMID:[33555128](https://www.ncbi.nlm.nih.gov/pubmed/33555128)

54. Slade T, Grove R, Burgess P. Kessler Psychological Distress Scale: Normative data from the 2007 Australian national survey of mental health and wellbeing. Australian and New Zealand journal of psychiatry journals.sagepub.com; 2011 Apr;45(4):308–316. PMID:[21332432](https://www.ncbi.nlm.nih.gov/pubmed/21332432)

55. Sunderland M, Mahoney A, Andrews G. Investigating the factor structure of the Kessler psychological distress scale in community and clinical samples of the Australian population. Journal of psychopathology and behavioral assessment Springer Science; Business Media LLC; 2012 Jun;34(2):253–259. doi: [10.1007/s10862-012-9276-7](https://doi.org/10.1007/s10862-012-9276-7)

56. Stallman HM. Psychological distress in university students: A comparison with general population data. Australian psychologist 2010;45(4):249–257. doi: [10.1080/00050067.2010.482109](https://doi.org/10.1080/00050067.2010.482109)

57. Cohen S, Kamarck T, Mermelstein R. A global measure of perceived stress. Journal of health and social behavior JSTOR; 1983 Dec;24(4):385–396. PMID:[6668417](https://www.ncbi.nlm.nih.gov/pubmed/6668417)

58. Cohen S. Perceived stress in a probability sample of the United States. The social psychology of health 1988;251:31–67. Available from: <https://psycnet.apa.org/fulltext/1988-98838-002.pdf>

59. Ezzati A, Jiang J, Katz MJ, Sliwinski MJ, Zimmerman ME, Lipton RB. Validation of the Perceived Stress Scale in a community sample of older adults. International journal of geriatric psychiatry 2014 Jun;29(6):645–652. PMID:[24302253](https://www.ncbi.nlm.nih.gov/pubmed/24302253)

60. Lee E-H. Review of the psychometric evidence of the Perceived Stress Scale. Asian nursing research 2012 Dec;6(4):121–127. PMID:[25031113](https://www.ncbi.nlm.nih.gov/pubmed/25031113)

61. Mitchell AM, Crane PA, Kim Y. Perceived stress in survivors of suicide: Psychometric properties of the Perceived Stress Scale. Research in nursing & health 2008 Dec;31(6):576–585. PMID:[18449942](https://www.ncbi.nlm.nih.gov/pubmed/18449942)

62. Wiriyakijja P, Porter S, Fedele S, Hodgson T, McMillan R, Shephard M, Ni Riordain R. Validation of the HADS and PSS-10 and psychological status in patients with oral lichen planus. Oral diseases 2020 Jan;26(1):96–110. PMID:[31650646](https://www.ncbi.nlm.nih.gov/pubmed/31650646)

63. Anwer S, Manzar MD, Alghadir AH, Salahuddin M, Abdul Hameed U. Psychometric analysis of the Perceived Stress Scale among healthy university students. Neuropsychiatric disease and treatment 2020 Oct;16:2389–2396. PMID:[33116538](https://www.ncbi.nlm.nih.gov/pubmed/33116538)

64. Chapman CB, Wilson SG, Wilson DI, Dunkley MK. National survey of pharmacists, intern pharmacists and pharmacy students in Australia: The nature and extent of stress and well-being. International journal of pharmacy practice 2020 Aug;28(4):355–361. PMID:[32101362](https://www.ncbi.nlm.nih.gov/pubmed/32101362)

65. Zigmond AS, Snaith RP. The Hospital Anxiety and Depression Scale. Acta psychiatrica Scandinavica 1983 Jun;67(6):361–370. PMID:[6880820](https://www.ncbi.nlm.nih.gov/pubmed/6880820)

66. Bjelland I, Dahl AA, Haug TT, Neckelmann D. The validity of the Hospital Anxiety and Depression Scale: An updated literature review. Journal of psychosomatic research 2002 Feb;52(2):69–77. PMID:[11832252](https://www.ncbi.nlm.nih.gov/pubmed/11832252)

67. Crawford JR, Henry JD, Crombie C, Taylor EP. Normative data for the HADS from a large non-clinical sample. British journal of clinical psychology 2001 Nov;40(4):429–434. PMID:[11760618](https://www.ncbi.nlm.nih.gov/pubmed/11760618)

68. McPherson A, Martin CR. Is the Hospital Anxiety and Depression Scale (HADS) an appropriate screening tool for use in an alcohol-dependent population? Journal of clinical nursing 2011 Jun;20(11-12):1507–1517. PMID:[20955485](https://www.ncbi.nlm.nih.gov/pubmed/20955485)

69. Karimova G, Martin C. A psychometric evaluation of the Hospital Anxiety and Depression Scale during pregnancy. Psychology, health & medicine Informa UK Limited; 2003 Feb;8(1):89–103. PMID:[21888492](https://www.ncbi.nlm.nih.gov/pubmed/21888492)

70. Herrmann C. International experiences with the hospital anxiety and depression scale–A review of validation data and clinical results. Journal of psychosomatic research 1997 Jan;42(1):17–41. PMID:[9055211](https://www.ncbi.nlm.nih.gov/pubmed/9055211)

71. Cameron IM, Crawford JR, Lawton K, Reid IC. Psychometric comparison of PHQ-9 and HADS for measuring depression severity in primary care. The British journal of general practice 2008 Jan;58(546):32–36. PMID:[18186994](https://www.ncbi.nlm.nih.gov/pubmed/18186994)

72. Beck AT, Steer RA, Brown G. Beck Depression Inventory–II. Psychological assessment 1996; doi: [10.1037/t00742-000](https://doi.org/10.1037/t00742-000)

73. Dozois DJA, Dobson KS, Ahnberg JL. A psychometric evaluation of the Beck Depression Inventory–II. Psychological assessment US: American Psychological Association; 1998;10(2):83. doi: [10.1037/1040-3590.10.2.83](https://doi.org/10.1037/1040-3590.10.2.83)

74. Wang Y-P, Gorenstein C. Psychometric properties of the Beck Depression Inventory-II: A comprehensive review. Revista brasileira de psiquiatria 2013 Oct;35(4):416–431. PMID:[24402217](https://www.ncbi.nlm.nih.gov/pubmed/24402217)

75. Carmody DP. Psychometric characteristics of the Beck Depression Inventory-II with college students of diverse ethnicity. International journal of psychiatry in clinical practice 2005;9(1):22–28. PMID:[24945333](https://www.ncbi.nlm.nih.gov/pubmed/24945333)

76. Whisman MA, Richardson ED. Normative data on the beck depression inventory–second edition (BDI-II) in college students. Journal of clinical psychology 2015 Sep;71(9):898–907. PMID:[25950150](https://www.ncbi.nlm.nih.gov/pubmed/25950150)

77. Diener E, Emmons RA, Larsen RJ, Griffin S. The Satisfaction With Life Scale. Journal of personality assessment 1985 Feb;49(1):71–75. PMID:[16367493](https://www.ncbi.nlm.nih.gov/pubmed/16367493)

78. Agteren J van, Ali K, Fassnacht DB, Iasiello M, Furber G, Howard A, Woodyatt L, Musker M, Kyrios M. Testing the Differential Impact of an Internet-Based Mental Health Intervention on Outcomes of Well-being and Psychological Distress During COVID-19: Uncontrolled Intervention Study. JMIR mental health 2021 Sep;8(9):e28044. PMID:[34357876](https://www.ncbi.nlm.nih.gov/pubmed/34357876)

79. Beuningen J van. The Satisfaction With Life Scale examining construct validity. Den Haag/Heerlen: Statistics Netherlands; 2012.

80. Pavot W, Diener E. Review of the Satisfaction With Life Scale. Psychological assessment 1993;5(2):164–172. doi: [10.1037/1040-3590.5.2.164](https://doi.org/10.1037/1040-3590.5.2.164)

81. Pavot W, Diener E, Colvin CR, Sandvik E. Further validation of the Satisfaction with Life Scale: Evidence for the cross-method convergence of well-being measures. Journal of personality assessment 1991 Aug;57(1):149–161. PMID:[1920028](https://www.ncbi.nlm.nih.gov/pubmed/1920028)

82. Pavot W, Diener E. The Satisfaction With Life Scale and the emerging construct of life satisfaction. The journal of positive psychology Routledge; 2008 Apr;3(2):137–152. doi: [10.1080/17439760701756946](https://doi.org/10.1080/17439760701756946)

83. Hagell P, Smith S. A psychometric comparison of two carer quality of life questionnaires in Huntington’s disease: Implications for neurodegenerative disorders. Journal of Huntington’s disease 2013;2(3):315–322. PMID:[25062679](https://www.ncbi.nlm.nih.gov/pubmed/25062679)

84. Rosengren L, Jonasson SB, Brogårdh C, Lexell J. Psychometric properties of the Satisfaction With Life Scale in Parkinson’s disease. Acta neurologica Scandinavica 2015 Sep;132(3):164–170. PMID:[25639961](https://www.ncbi.nlm.nih.gov/pubmed/25639961)

85. Cheung F, Lucas RE. Assessing the validity of single-item life satisfaction measures: Results from three large samples. Quality of life research 2014 Dec;23(10):2809–2818. PMID:[24890827](https://www.ncbi.nlm.nih.gov/pubmed/24890827)

86. Radloff LS. The CES-D scale: A self-report depression scale for research in the general population. Applied psychological measurement SAGE Publications Inc; 1977 Jun;1(3):385–401. doi: [10.1177/014662167700100306](https://doi.org/10.1177/014662167700100306)

87. Mongrain M, Barnes C, Barnhart R, Zalan LB. Acts of kindness reduce depression in individuals low on agreeableness. Translational Issues in Psychological Science 2018 Sep;4(3):323–334. doi: [10.1037/tps0000168](https://doi.org/10.1037/tps0000168)

88. Vilagut G, Forero CG, Barbaglia G, Alonso J. Screening for depression in the general population with the Center for Epidemiologic Studies Depression (CES-D): A systematic review with meta-analysis. PLOS One 2016 May;11(5):e0155431. PMID:[27182821](https://www.ncbi.nlm.nih.gov/pubmed/27182821)

89. Hann D, Winter K, Jacobsen P. Measurement of depressive symptoms in cancer patients: Evaluation of the Center for Epidemiological Studies Depression Scale (CES-D). Journal of psychosomatic research 1999 May;46(5):437–443. PMID:[10404478](https://www.ncbi.nlm.nih.gov/pubmed/10404478)

90. Wilson RS, Mendes De Leon CF, Bennett DA, Bienias JL, Evans DA. Depressive symptoms and cognitive decline in a community population of older persons. Journal of neurology, neurosurgery, and psychiatry BMJ Publishing Group Ltd; 2004 Jan;75(1):126–129. PMID:[14707321](https://www.ncbi.nlm.nih.gov/pubmed/14707321)

91. Christensen H, Batterham PJ, Grant JB, Griffiths KM, Mackinnon AJ. A population study comparing screening performance of prototypes for depression and anxiety with standard scales. BMC medical research methodology 2011 Nov;11:154. PMID:[22103584](https://www.ncbi.nlm.nih.gov/pubmed/22103584)

92. Tennant R, Hiller L, Fishwick R, Platt S, Joseph S, Weich S, Parkinson J, Secker J, Stewart-Brown S. The Warwick-Edinburgh Mental Well-being Scale (WEMWBS): Development and UK validation. Health and quality of life outcomes 2007 Nov;5:63. PMID:[18042300](https://www.ncbi.nlm.nih.gov/pubmed/18042300)

93. Lloyd K, Devine P. Psychometric properties of the Warwick-Edinburgh Mental Well-being Scale (WEMWBS) in northern Ireland. Journal of mental health 2012 Jun;21(3):257–263. PMID:[22574955](https://www.ncbi.nlm.nih.gov/pubmed/22574955)

94. Maheswaran H, Weich S, Powell J, Stewart-Brown S. Evaluating the responsiveness of the Warwick Edinburgh Mental Well-Being Scale (WEMWBS): Group and individual level analysis. Health and quality of life outcomes 2012 Dec;10:156. PMID:[23270465](https://www.ncbi.nlm.nih.gov/pubmed/23270465)

95. Kroenke K, Strine TW, Spitzer RL, Williams JBW, Berry JT, Mokdad AH. The PHQ-8 as a measure of current depression in the general population. Journal of affective disorders 2009 Apr;114(1-3):163–173. PMID:[18752852](https://www.ncbi.nlm.nih.gov/pubmed/18752852)

96. Dancyger C, Kelleher K, Barrington M. A patient centred approach to measuring outcomes in psycho-oncology services: The PHQ-8 as a viable alternative to the PHQ-9. Psycho-Oncology 2022 Mar;31(3):548–550. PMID:[34792261](https://www.ncbi.nlm.nih.gov/pubmed/34792261)

97. Thakur E, Recober A, Turvey C, Dindo LN. Benefits of an on-line migraine education video for patients with co-occurring migraine and depression. Journal of psychosomatic research 2018 Sep;112:47–52. PMID:[30097135](https://www.ncbi.nlm.nih.gov/pubmed/30097135)

98. Jin H, Wu S. Text messaging as a screening tool for depression and related conditions in underserved, predominantly minority safety net primary care patients: Validity study. Journal of medical Internet research 2020 Mar;22(3):e17282. PMID:[32213473](https://www.ncbi.nlm.nih.gov/pubmed/32213473)

99. Wu Y, Levis B, Riehm KE, Saadat N, Levis AW, Azar M, Rice DB, Boruff J, Cuijpers P, Gilbody S, Ioannidis JPA, Kloda LA, McMillan D, Patten SB, Shrier I, Ziegelstein RC, Akena DH, Arroll B, Ayalon L, Baradaran HR, Baron M, Bombardier CH, Butterworth P, Carter G, Chagas MH, Chan JCN, Cholera R, Conwell Y, Man-van Ginkel JM de, Fann JR, Fischer FH, Fung D, Gelaye B, Goodyear-Smith F, Greeno CG, Hall BJ, Harrison PA, Härter M, Hegerl U, Hides L, Hobfoll SE, Hudson M, Hyphantis T, Inagaki M, Jetté N, Khamseh ME, Kiely KM, Kwan Y, Lamers F, Liu S-I, Lotrakul M, Loureiro SR, Löwe B, McGuire A, Mohd-Sidik S, Munhoz TN, Muramatsu K, Osório FL, Patel V, Pence BW, Persoons P, Picardi A, Reuter K, Rooney AG, Santos IS, Shaaban J, Sidebottom A, Simning A, Sung S, Tan PLL, Turner A, Weert HC van, White J, Whooley MA, Winkley K, Yamada M, Benedetti A, Thombs BD. Equivalency of the diagnostic accuracy of the PHQ-8 and PHQ-9: a systematic review and individual participant data meta-analysis. Psychological medicine Cambridge University Press; 2020 Jun;50(8):1368–1380. doi: [10.1017/S0033291719001314](https://doi.org/10.1017/S0033291719001314)

100. Watson D, Clark LA, Tellegen A. Development and validation of brief measures of positive and negative affect: The PANAS scales. Journal of personality and social psychology 1988 Jun;54(6):1063–1070. PMID:[3397865](https://www.ncbi.nlm.nih.gov/pubmed/3397865)

101. Crawford JR, Henry JD. The Positive and Negative Affect Schedule (PANAS): Construct validity, measurement properties and normative data in a large non-clinical sample. British journal of clinical psychology 2004;43(3):245–265. doi: [10.1348/0144665031752934](https://doi.org/10.1348/0144665031752934)

102. Rush AJ, Gullion CM, Basco MR, Jarrett RB, Trivedi MH. The Inventory of Depressive Symptomatology (IDS): Psychometric properties. Psychological medicine 1996 May;26(3):477–486. PMID:[8733206](https://www.ncbi.nlm.nih.gov/pubmed/8733206)

103. Rush AJ, Trivedi MH, Ibrahim HM, Carmody TJ, Arnow B, Klein DN, Markowitz JC, Ninan PT, Kornstein S, Manber R, Thase ME, Kocsis JH, Keller MB. The 16-Item Quick Inventory of Depressive Symptomatology (QIDS), clinician rating (QIDS-C), and self-report (QIDS-SR): A psychometric evaluation in patients with chronic major depression. Biological psychiatry 2003 Sep;54(5):573–583. PMID:[12946886](https://www.ncbi.nlm.nih.gov/pubmed/12946886)

104. Jordan DG, Collins AC, Dunaway MG, Kilgore J, Winer ES. Negative affect interference and fear of happiness are independently associated with depressive symptoms. Journal of clinical psychology 2021 Mar;77(3):646–660. PMID:[33078847](https://www.ncbi.nlm.nih.gov/pubmed/33078847)

105. Reilly TJ, MacGillivray SA, Reid IC, Cameron IM. Psychometric properties of the 16-item Quick Inventory of Depressive Symptomatology: A systematic review and meta-analysis. Journal of psychiatric research Elsevier; 2015 Jan;60:132–140. PMID:[25300442](https://www.ncbi.nlm.nih.gov/pubmed/25300442)

106. Trivedi MH, Rush AJ, Ibrahim HM, Carmody TJ, Biggs MM, Suppes T, Crismon ML, Shores-Wilson K, Toprac MG, Dennehy EB, Witte B, Kashner TM. The Inventory of Depressive Symptomatology, Clinician Rating (IDS-C) and Self-Report (IDS-SR), and the Quick Inventory of Depressive Symptomatology, Clinician Rating (QIDS-C) and Self-Report (QIDS-SR) in public sector patients with mood disorders: A psychometric evaluation. Psychological medicine Cambridge University Press (CUP); 2004 Jan;34(1):73–82. PMID:[14971628](https://www.ncbi.nlm.nih.gov/pubmed/14971628)

107. Lamoureux BE, Linardatos E, Fresco DM, Bartko D, Logue E, Milo L. Using the QIDS-SR16 to identify major depressive disorder in primary care medical patients. Behavior therapy 2010 Sep;41(3):423–431. PMID:[20569790](https://www.ncbi.nlm.nih.gov/pubmed/20569790)

108. Cameron IM, Crawford JR, Cardy AH, Toit SW du, Lawton K, Hay S, Mitchell K, Sharma S, Shivaprasad S, Winning S, Reid IC. Psychometric properties of the Quick Inventory of Depressive Symptomatology (QIDS-SR) in UK primary care. Journal of psychiatric research 2013 May;47(5):592–598. PMID:[23419617](https://www.ncbi.nlm.nih.gov/pubmed/23419617)

109. Saunders JB, Aasland OG, Babor TF, Fuente JR de la, Grant M. Development of the alcohol use disorders identification test (AUDIT): WHO collaborative project on early detection of persons with harmful alcohol consumption–II. Addiction 1993 Jun;88(6):791–804. PMID:[8329970](https://www.ncbi.nlm.nih.gov/pubmed/8329970)

110. Babor TF, Higgins-Biddle JC, Saunders JB, Monteiro MG, Others. The Alcohol Use Disorders Identification Test. World Health Organization Geneva; 2001. Available from: <http://www.psiholocator.com/images/who_msd_msb_016a.pdf>

111. Reinert DF, Allen JP. The alcohol use disorders identification test (AUDIT): A review of recent research. Alcoholism: clinical and experimental research Wiley; 2002 Feb;26(2):272–279. doi: [10.1111/j.1530-0277.2002.tb02534.x](https://doi.org/10.1111/j.1530-0277.2002.tb02534.x)

112. Maisto SA, Conigliaro J, McNeil M, Kraemer K, Kelley ME. An empirical investigation of the factor structure of the AUDIT. Psychological assessment American Psychological Association (APA); 2000;12(3):346–353. doi: [10.1037/1040-3590.12.3.346](https://doi.org/10.1037/1040-3590.12.3.346)

113. Allen JP, Litten RZ, Fertig JB, Babor T. A review of research on the Alcohol Use Disorders Identification Test (AUDIT). Alcoholism: Clinical and Experimental Research Wiley; 1997 Jun;21(4):613–619. doi: [10.1111/j.1530-0277.1997.tb03811.x](https://doi.org/10.1111/j.1530-0277.1997.tb03811.x)

114. Clements R. A critical evaluation of several alcohol screening instruments using the CIDI-SAM as a criterion measure. Alcoholism: clinical and experimental research 1998 Aug;22(5):985–993. PMID:[9726267](https://www.ncbi.nlm.nih.gov/pubmed/9726267)

115. Peng C-Z, Wilsnack RW, Kristjanson AF, Benson P, Wilsnack SC. Gender differences in the factor structure of the Alcohol Use Disorders Identification Test in multinational general population surveys. Drug and alcohol dependence Elsevier BV; 2012 Jul;124(1-2):50–56. PMID:[22236536](https://www.ncbi.nlm.nih.gov/pubmed/22236536)

116. Fairburn CG, Beglin SJ. Assessment of eating disorders: Interview or self-report questionnaire? The International journal of eating disorders 1994 Dec;16(4):363–370. PMID:[7866415](https://www.ncbi.nlm.nih.gov/pubmed/7866415)

117. Mond JM, Hay PJ, Rodgers B, Owen C. Eating Disorder Examination Questionnaire (EDE-Q): Norms for young adult women. Behaviour research and therapy Elsevier; 2006 Jan;44(1):53–62. PMID:[16301014](https://www.ncbi.nlm.nih.gov/pubmed/16301014)

118. Luce KH, Crowther JH. The reliability of the Eating Disorder Examination-Self-Report Questionnaire version (EDE-Q). The International journal of eating disorders 1999 Apr;25(3):349–351. PMID:[10192002](https://www.ncbi.nlm.nih.gov/pubmed/10192002)

119. Berg KC, Peterson CB, Frazier P, Crow SJ. Psychometric evaluation of the eating disorder examination and eating disorder examination-questionnaire: A systematic review of the literature. International journal of eating disorders 2012 Apr;45(3):428–438. PMID:[21744375](https://www.ncbi.nlm.nih.gov/pubmed/21744375)

120. Mond JM, Hay PJ, Rodgers B, Owen C, Beumont PJV. Validity of the Eating Disorder Examination Questionnaire (EDE-Q) in screening for eating disorders in community samples. Behaviour research and therapy 2004 May;42(5):551–567. PMID:[15033501](https://www.ncbi.nlm.nih.gov/pubmed/15033501)

121. Rosen JC, Vara L, Wendt S, Leitenberg H. Validity studies of the eating disorder examination. International journal of eating disorders 1990 Sep;9(5):519–528. doi: [10.1002/1098-108X(199009)9:5<519::AID-EAT2260090507>3.0.CO;2-K](https://doi.org/10.1002/1098-108X(199009)9:5%3c519::AID-EAT2260090507%3e3.0.CO;2-K)

122. Jennings KM, Phillips KE. Eating disorder examination-questionnaire (EDE-Q): Norms for clinical sample of female adolescents with anorexia nervosa. Archives of psychiatric nursing 2017 Dec;31(6):578–581. PMID:[29179824](https://www.ncbi.nlm.nih.gov/pubmed/29179824)

123. Weiss DS, Marmar CR. The Impact of Event Scale—Revised. In: Wilson JP, editor. Assessing psychological trauma and PTSD New York, NY, US: The Guilford Press, xiv; 1997. p. 399–411. Available from: <https://psycnet.apa.org/fulltext/1997-97162-013.pdf>

124. Weiss D. The Impact of Event Scale - Revised. In: John P. Wilson and Terence M. Keane, editor. Assessing psychological trauma and PTSD, second edition 2004.

125. Creamer M, Bell R, Failla S. Psychometric properties of the Impact of Event Scale - Revised. Behaviour research and therapy 2003 Dec;41(12):1489–1496. PMID:[14705607](https://www.ncbi.nlm.nih.gov/pubmed/14705607)

126. Adkins JW, Weathers FW, McDevitt-Murphy M, Daniels JB. Psychometric properties of seven self-report measures of posttraumatic stress disorder in college students with mixed civilian trauma exposure. Journal of anxiety disorders 2008 Dec;22(8):1393–1402. PMID:[18436427](https://www.ncbi.nlm.nih.gov/pubmed/18436427)

127. Beck JG, Grant DM, Read JP, Clapp JD, Coffey SF, Miller LM, Palyo SA. The impact of event scale-revised: Psychometric properties in a sample of motor vehicle accident survivors. Journal of anxiety disorders Elsevier BV; 2008 Jan;22(2):187–198. PMID:[17369016](https://www.ncbi.nlm.nih.gov/pubmed/17369016)

128. Ware JE. SF-36 physical and mental health summary scores. Boston: The Health Institute; 1994. ISBN:9781891810008

129. Ware JE, Sherbourne CD. The MOS 36-item short-form health survey (SF-36). I. Conceptual framework and item selection. Medical care Lippincott Williams & Wilkins; 1992 Jun;30(6):473–483. PMID:[1593914](https://www.ncbi.nlm.nih.gov/pubmed/1593914)

130. Butterworth P, Crosier T. The validity of the SF-36 in an Australian National Household Survey: Demonstrating the applicability of the Household Income and Labour Dynamics in Australia (HILDA) Survey to examination of health inequalities. BMC public health Springer Nature; 2004 Oct;4(1):44. PMID:[15469617](https://www.ncbi.nlm.nih.gov/pubmed/15469617)

131. Brazier JE, Harper R, Jones NM, O’Cathain A, Thomas KJ, Usherwood T, Westlake L. Validating the SF-36 health survey questionnaire: New outcome measure for primary care. BMJ 1992 Jul;305(6846):160–164. PMID:[1285753](https://www.ncbi.nlm.nih.gov/pubmed/1285753)

132. Jenkinson C, Wright L, Coulter A. Criterion validity and reliability of the SF-36 in a population sample. Quality of life research Springer Nature; 1994 Feb;3(1):7–12. PMID:[8142947](https://www.ncbi.nlm.nih.gov/pubmed/8142947)

133. Stansfeld SA, Roberts R, Foot SP. Assessing the validity of the SF-36 General Health Survey. Quality of life research Springer; 1997 Apr;6(3):217–224. PMID:[9226979](https://www.ncbi.nlm.nih.gov/pubmed/9226979)

134. Garratt AM, Ruta DA, Abdalla MI, Russell IT. SF 36 health survey questionnaire: II. Responsiveness to changes in health status in four common clinical conditions. BMJ Quality and Safety 1994 Dec;3(4):186–192. PMID:[10140232](https://www.ncbi.nlm.nih.gov/pubmed/10140232)

135. Katz JN, Larson MG, Phillips CB, Fossel AH, Liang MH. Comparative measurement sensitivity of short and longer health status instruments. Medical care 1992 Oct;30(10):917–925. PMID:[1405797](https://www.ncbi.nlm.nih.gov/pubmed/1405797)

136. Thumboo J, Fong KY, Machin D, Chan SP, Leon KH, Feng PH, Thio ST, Boe ML. A community-based study of scaling assumptions and construct validity of the English (UK) and Chinese (HK) SF-36 in Singapore. Quality of life research Springer; 2001;10(2):175–188. PMID:[11642688](https://www.ncbi.nlm.nih.gov/pubmed/11642688)

137. Jenkinson C, Coulter A, Wright L. Short form 36 (SF36) health survey questionnaire: Normative data for adults of working age. BMJ 1993 May;306(6890):1437–1440. PMID:[8518639](https://www.ncbi.nlm.nih.gov/pubmed/8518639)

138. Houck PR, Spiegel DA, Shear MK, Rucci P. Reliability of the self-report version of the panic disorder severity scale. Depression and anxiety 2002;15(4):183–185. PMID:[12112724](https://www.ncbi.nlm.nih.gov/pubmed/12112724)

139. Hadjistavropoulos HD, Schneider LH, Mehta S, Karin E, Dear BF, Titov N. Preference trial of internet-delivered cognitive behaviour therapy comparing standard weekly versus optional weekly therapist support. Journal of anxiety disorders 2019 Apr;63:51–60. PMID:[30844613](https://www.ncbi.nlm.nih.gov/pubmed/30844613)

140. Shear MK, Brown TA, Barlow DH, Money R, Sholomskas DE, Woods SW, Gorman JM, Papp LA. Multicenter collaborative panic disorder severity scale. American journal of psychiatry 1997 Nov;154(11):1571–1575. PMID:[9356566](https://www.ncbi.nlm.nih.gov/pubmed/9356566)

141. Newby JM, Mewton L, Williams AD, Andrews G. Effectiveness of transdiagnostic internet cognitive behavioural treatment for mixed anxiety and depression in primary care. Journal of affective disorders 2014 Aug;165:45–52. PMID:[24882176](https://www.ncbi.nlm.nih.gov/pubmed/24882176)

142. Warttig SL, Forshaw MJ, South J, White AK. New, normative, English-sample data for the Short Form Perceived Stress Scale (PSS-4). Journal of health psychology 2013 Dec;18(12):1617–1628. PMID:[24155195](https://www.ncbi.nlm.nih.gov/pubmed/24155195)

143. Wu SM, Amtmann D. Psychometric evaluation of the Perceived Stress Scale in multiple sclerosis. ISRN Rehabilitation Hindawi Limited; 2013 Dec;2013:1–9. doi: [10.1155/2013/608356](https://doi.org/10.1155/2013/608356)

144. Goldberg SB, Imhoff-Smith T, Bolt DM, Wilson-Mendenhall CD, Dahl CJ, Davidson RJ, Rosenkranz MA. Testing the efficacy of a multicomponent, self-guided, smartphone-based meditation app: Three-armed randomized controlled trial. JMIR mental health 2020 Nov;7(11):e23825. PMID:[33245288](https://www.ncbi.nlm.nih.gov/pubmed/33245288)

145. Ribeiro Santiago PH, Nielsen T, Smithers LG, Roberts R, Jamieson L. Measuring stress in Australia: Validation of the Perceived Stress Scale (PSS-14) in a national sample. Health and quality of life outcomes 2020 Apr;18(1):100. PMID:[32295596](https://www.ncbi.nlm.nih.gov/pubmed/32295596)

146. Ribeiro Santiago PH. Psychometric properties of the Perceived Stress Scale (PSS), Social Support Scale (SSS) and Sense of Personal Control Scale (SPCS) in Aboriginal Australian populations [PhD thesis]. 2020. Available from: <https://digital.library.adelaide.edu.au/dspace/handle/2440/124410>

147. Skevington SM, Lotfy M, O’Connell KA. The World Health Organization’s WHOQOL-BREF quality of life assessment: Psychometric properties and results of the international field trial. A report from the WHOQOL Group. Quality of life research 2004 Mar;13(2):299–310. PMID:[15085902](https://www.ncbi.nlm.nih.gov/pubmed/15085902)

148. Skevington SM, McCrate FM. Expecting a good quality of life in health: Assessing people with diverse diseases and conditions using the WHOQOL-BREF. Health expectations 2012;15(1):49–62. doi: [10.1111/j.1369-7625.2010.00650.x](https://doi.org/10.1111/j.1369-7625.2010.00650.x)

149. Suárez L, Tay B, Abdullah F. Psychometric properties of the World Health Organization WHOQOL-BREF Quality of Life assessment in Singapore. Quality of life research 2018 Nov;27(11):2945–2952. PMID:[30046975](https://www.ncbi.nlm.nih.gov/pubmed/30046975)

150. EuroQol Group. EuroQol–a new facility for the measurement of health-related quality of life. Health policy 1990 Dec;16(3):199–208. PMID:[10109801](https://www.ncbi.nlm.nih.gov/pubmed/10109801)

151. Khanna R, Jariwala K, Bentley JP. Psychometric properties of the EuroQol Five Dimensional Questionnaire (EQ-5D-3L) in caregivers of autistic children. Quality of life research Springer; 2013 Dec;22(10):2909–2920. PMID:[23615959](https://www.ncbi.nlm.nih.gov/pubmed/23615959)

152. Zakershahrak M, Ribeiro Santiago PH, Sethi S, Haag D, Jamieson L, Brennan D. Psychometric properties of the EQ-5D-3L in South Australia: A multi-method non-preference-based validation study. Current medical research and opinion 2022 May;38(5):673–685. PMID:[35060425](https://www.ncbi.nlm.nih.gov/pubmed/35060425)

153. Dolan P, Grudex C, Kind P, Williams A. The measurement and valuation of health: First report on the main survey. The MVH Group; 1994. Available from: <https://www.york.ac.uk/media/che/documents/reports/MVH%20First%20Report.pdf>

154. Brazier J, Jones N, Kind P. Testing the validity of the Euroqol and comparing it with the SF-36 health survey questionnaire. Quality of life research 1993 Jun;2(3):169–180. PMID:[8401453](https://www.ncbi.nlm.nih.gov/pubmed/8401453)

155. Janssen MF, Pickard AS, Golicki D, Gudex C, Niewada M, Scalone L, Swinburn P, Busschbach J. Measurement properties of the EQ-5D-5L compared to the EQ-5D-3L across eight patient groups: A multi-country study. Quality of life research 2013 Sep;22(7):1717–1727. PMID:[23184421](https://www.ncbi.nlm.nih.gov/pubmed/23184421)

156. Keyes CLM. Mental illness and/or mental health? Investigating axioms of the complete state model of health. Journal of consulting and clinical psychology 2005;73(3):539–548. doi: [10.1037/0022-006X.73.3.539](https://doi.org/10.1037/0022-006X.73.3.539)

157. Levin ME, Haeger JA, Pierce BG, Twohig MP. Web-based acceptance and commitment therapy for mental health problems in college students: A randomized controlled trial. Behavior modification 2017 Jan;41(1):141–162. PMID:[27440189](https://www.ncbi.nlm.nih.gov/pubmed/27440189)

158. Pir S, Hashemi L, Gulliver P, Fanslow J. Psychometric evaluation of the Mental Health Continuum-Short Form (MHC-SF) in a New Zealand context – A confirmatory factor analysis. Current psychology Springer Science; Business Media LLC; 2021;42(13):11170–11183. doi: [10.1007/s12144-021-02401-3](https://doi.org/10.1007/s12144-021-02401-3)

159. Orpana H, Vachon J, Dykxhoorn J, Jayaraman G. Measuring positive mental health in Canada: Construct validation of the Mental Health Continuum-Short Form. Health promotion and chronic disease prevention in Canada Health Promotion; Chronic Disease Prevention Branch (HPCDP) Public Health Agency of Canada; 2017 Apr;37(4):123–130. PMID:[28402801](https://www.ncbi.nlm.nih.gov/pubmed/28402801)

160. Iasiello M, Agteren J van, Schotanus-Dijkstra M, Lo L, Fassnacht DB, Westerhof GJ. Assessing mental wellbeing using the Mental Health Continuum—Short Form: A systematic review and meta-analytic structural equation modelling. Clinical Psychology: Science and Practice 2022 Mar;29(4):442–456. doi: [10.1037/cps0000074](https://doi.org/10.1037/cps0000074)

161. Weiss LA, Westerhof GJ, Bohlmeijer ET. Can we increase psychological well-being? The effects of interventions on psychological well-being: A meta-analysis of randomized controlled trials. PLOS One 2016 Jun;11(6):e0158092. PMID:[27328124](https://www.ncbi.nlm.nih.gov/pubmed/27328124)

162. Hides L, Quinn C, Stoyanov S, Cockshaw W, Mitchell T, Kavanagh DJ. Is the mental wellbeing of young Australians best represented by a single, multidimensional or bifactor model? Psychiatry research 2016 Jul;241:1–7. PMID:[27152903](https://www.ncbi.nlm.nih.gov/pubmed/27152903)

163. Spielberger CD. State-Trait Anxiety Inventory for adults. 1983; doi: [10.1037/t06496-000](https://doi.org/10.1037/t06496-000)

164. American Psychological Association. The State-Trait Anxiety Inventory (STAI). <https://www.apa.org/pi/about/publications/caregivers/practice-settings/assessment/tools/trait-state>; 2011.

165. Barnes LLB, Harp D, Jung WS. Reliability generalization of scores on the Spielberger State-Trait Anxiety Inventory. Educational and psychological measurement SAGE Publications; 2002 Aug;62(4):603–618. doi: [10.1177/0013164402062004005](https://doi.org/10.1177/0013164402062004005)

166. Shahid A, Wilkinson K, Marcu S, Shapiro CM. STOP, THAT and one hundred other sleep scales. Springer Science & Business Media; 2012. doi: [10.1007/978-1-4419-9893-4](https://doi.org/10.1007/978-1-4419-9893-4)ISBN:9781441998927

167. Balsamo M, Romanelli R, Innamorati M, Ciccarese G, Carlucci L, Saggino A. The state-trait anxiety inventory: Shadows and lights on its construct validity. Journal of psychopathology and behavioral assessment 2013 Dec;35(4):475–486. doi: [10.1007/s10862-013-9354-5](https://doi.org/10.1007/s10862-013-9354-5)

168. Julian LJ. Measures of anxiety: State-Trait Anxiety Inventory (STAI), Beck Anxiety Inventory (BAI), and Hospital Anxiety and Depression Scale-Anxiety (HADS-A). Arthritis care & research 2011 Nov;63(S11):S467–S472. PMID:[22588767](https://www.ncbi.nlm.nih.gov/pubmed/22588767)

169. Santangelo G, Sacco R, Siciliano M, Bisecco A, Muzzo G, Docimo R, De Stefano M, Bonavita S, Lavorgna L, Tedeschi G, Trojano L, Gallo A. Anxiety in multiple sclerosis: Psychometric properties of the State-Trait Anxiety Inventory. Acta neurologica Scandinavica 2016 Dec;134(6):458–466. PMID:[27219913](https://www.ncbi.nlm.nih.gov/pubmed/27219913)

170. Zamperoni V, Breedvelt J, Kousoulis A, South E, Uphoff E, Gilbody S, Bockting C, Churchill R. A systematic review of mental health measurement scales for the assessment of the effects of mental health prevention interventions. PROSPERO 2018; Available from: <https://www.crd.york.ac.uk/prospero/display_record.php?RecordID=95519>

171. Martin-Key NA, Spadaro B, Funnell E, Barker EJ, Schei TS, Tomasik J, Bahn S. The current state and validity of digital assessment tools for psychiatry: Systematic review. JMIR mental health 2022 Mar;9(3):e32824. PMID:[35353053](https://www.ncbi.nlm.nih.gov/pubmed/35353053)

172. Breedvelt JJF, Zamperoni V, South E, Uphoff EP, Gilbody S, Bockting CLH, Churchill R, Kousoulis AA. A systematic review of mental health measurement scales for evaluating the effects of mental health prevention interventions. European journal of public health 2020 Jun;30(3):510–516. PMID:[32236548](https://www.ncbi.nlm.nih.gov/pubmed/32236548)

173. Elsman EBM, Mokkink LB, Terwee CB, Beaton D, Gagnier JJ, Tricco AC, Baba A, Butcher NJ, Smith M, Hofstetter C, Aiyegbusi OL, Berardi A, Farmer J, Haywood KL, Krause KR, Markham S, Mayo-Wilson E, Mehdipour A, Ricketts J, Szatmari P, Touma Z, Moher D, Offringa M. Guideline for reporting systematic reviews of outcome measurement instruments (OMIs): PRISMA-COSMIN for OMIs 2024. Quality of life research Springer Science; Business Media LLC; 2024 Aug;33(8):2029–2046. PMID:[38980635](https://www.ncbi.nlm.nih.gov/pubmed/38980635)

174. Coulacoglou C, Saklofske DH. Psychometrics and psychological assessment: Principles and applications. Academic Press; 2017. ISBN:9780128024904

175. Schmitter-Edgecombe M, Sumida C, Cook DJ. Bridging the gap between performance-based assessment and self-reported everyday functioning: An ecological momentary assessment approach. The clinical neuropsychologist Informa UK Limited; 2020 May;34(4):678–699. PMID:[32189568](https://www.ncbi.nlm.nih.gov/pubmed/32189568)

176. Collie A, Maruff P, Darby DG, McStephen M. The effects of practice on the cognitive test performance of neurologically normal individuals assessed at brief test-retest intervals. Journal of the International Neuropsychological Society Cambridge University Press; 2003 Mar;9(3):419–428. PMID:[12666766](https://www.ncbi.nlm.nih.gov/pubmed/12666766)

177. Hausknecht JP, Halpert JA, Di Paolo NT, Moriarty Gerrard MO. Retesting in selection: A meta-analysis of coaching and practice effects for tests of cognitive ability. Journal of applied psychology American Psychological Association (APA); 2007 Mar;92(2):373–385. PMID:[17371085](https://www.ncbi.nlm.nih.gov/pubmed/17371085)

178. Chen R, Calmasini C, Swinnerton K, Wang J, Haneuse S, Ackley SF, Hirst AK, Hayes-Larson E, George KM, Peterson R, Soh Y, Barnes LL, Mayeda ER, Gilsanz P, Mungas DM, Whitmer RA, Corrada MM, Glymour MM. Pragmatic approaches to handling practice effects in longitudinal cognitive aging research. Alzheimer’s & dementia John Wiley & Sons, Ltd; 2023 Sep;19(9):4028–4036. PMID:[37199336](https://www.ncbi.nlm.nih.gov/pubmed/37199336)

179. Achtyes ED, Halstead S, Smart L, Moore T, Frank E, Kupfer DJ, Gibbons R. Validation of computerized adaptive testing in an outpatient nonacademic setting: The VOCATIONS trial. Psychiatric services 2015 Oct;66(10):1091–1096. PMID:[26030317](https://www.ncbi.nlm.nih.gov/pubmed/26030317)

180. McAleavey AA. When (not) to rely on the reliable change index: A critical appraisal and alternatives to consider in clinical psychology. Clinical psychology American Psychological Association (APA); 2024 Apr;31(3):351–366. doi: [10.1037/cps0000203](https://doi.org/10.1037/cps0000203)

1. The decision to include all modalities was due to the scarcity of psychometric reviews and evaluation studies for mental health instruments administered digitally in the general population. For e.g., one author (ZHK) searched the COSMIN database (https://database.cosmin.nl/) for systematic reviews of the PHQ-9 (one of the widely used self-report instruments) but failed to find any review that met the three criteria we defined in our review. [↑](#footnote-ref-1)
